# Increased Duration of Paid Maternity Leave Lowers Infant Mortality in Low- and Middle-Income Countries: A Quasi-Experimental Study

**DOI:** 10.1371/journal.pmed.1001985

**Published:** 2016-03-29

**Authors:** Arijit Nandi, Mohammad Hajizadeh, Sam Harper, Alissa Koski, Erin C. Strumpf, Jody Heymann

**Affiliations:** 1 Institute for Health and Social Policy, McGill University, Montreal, Quebec, Canada; 2 Department of Epidemiology, Biostatistics, and Occupational Health, McGill University, Montreal, Quebec, Canada; 3 School of Health Administration, Dalhousie University, Halifax, Nova Scotia, Canada; 4 Department of Economics, McGill University, Montreal, Quebec, Canada; 5 Fielding School of Public Health, University of California, Los Angeles, Los Angeles, California, United States of America; Institute for Global Health, UNITED KINGDOM

## Abstract

**Background:**

Maternity leave reduces neonatal and infant mortality rates in high-income countries. However, the impact of maternity leave on infant health has not been rigorously evaluated in low- and middle-income countries (LMICs). In this study, we utilized a difference-in-differences approach to evaluate whether paid maternity leave policies affect infant mortality in LMICs.

**Methods and Findings:**

We used birth history data collected via the Demographic and Health Surveys to assemble a panel of approximately 300,000 live births in 20 countries from 2000 to 2008; these observational data were merged with longitudinal information on the duration of paid maternity leave provided by each country. We estimated the effect of an increase in maternity leave in the prior year on the probability of infant (<1 y), neonatal (<28 d), and post-neonatal (between 28 d and 1 y after birth) mortality. Fixed effects for country and year were included to control for, respectively, unobserved time-invariant confounders that varied across countries and temporal trends in mortality that were shared across countries. Average rates of infant, neonatal, and post-neonatal mortality over the study period were 55.2, 30.7, and 23.0 per 1,000 live births, respectively. Each additional month of paid maternity was associated with 7.9 fewer infant deaths per 1,000 live births (95% CI 3.7, 12.0), reflecting a 13% relative reduction. Reductions in infant mortality associated with increases in the duration of paid maternity leave were concentrated in the post-neonatal period. Estimates were robust to adjustment for individual, household, and country-level characteristics, although there may be residual confounding by unmeasured time-varying confounders, such as coincident policy changes.

**Conclusions:**

More generous paid maternity leave policies represent a potential instrument for facilitating early-life interventions and reducing infant mortality in LMICs and warrant further discussion in the post-2015 sustainable development agenda. From a policy planning perspective, further work is needed to elucidate the mechanisms that explain the benefits of paid maternity leave for infant mortality.

## Abstract 2

Nandi and colleagues, in a large study analyzing data from 20 countries, show that extended, paid maternity leave effectively reduces infant mortality in LMICs.

## Editors' Summary

**Background:**

In 1990, 12 million children—most of them living in low- and middle-income countries (LMICs)—died before their fifth birthday. Out of every 1,000 children born alive, 90 died before they were five years old. In 2000, world leaders set a target of reducing under-five mortality (deaths) to one-third of its 1990 level by 2015 as Millennium Development Goal 4 (MDG4); this goal, together with seven others, was designed to eradicate extreme poverty globally. Over the years, steady progress was made towards MDG4. Better delivery facilities and programs that encouraged breastfeeding, immunization, and other practices that improved the health of young children reduced the global under-five mortality rate. But, by 2015, the rate had only fallen to 43 deaths per 1,000 live births, and 5.9 million children under five died that year from preventable causes such as preterm birth complications, delivery complications, and infections. Nearly half of these deaths occurred among neonates (babies less than 28 days old); three-quarters of them occurred among infants (children less than 1 year old).

**Why Was This Study Done?:**

In high-income countries, paid leave from employment for mothers is associated with reduced neonatal and infant mortality. Many LMICs now have legislation granting paid maternity leave or gender-neutral parental leave. But does paid maternity leave have the same impact on infant health in LMICs as it does in high-income countries? In this quasi-experimental study, the researchers use the difference-in-differences statistical approach to investigate whether paid maternity leave policies affect infant mortality in LMICs. A quasi-experimental study uses observational data to compare outcomes in a group of people receiving an intervention (the treatment group) with outcomes in a group of people not receiving the intervention (control group); unlike a randomized controlled trial, these groups are not chosen at random. The difference-in-differences approach compares the average change over time in an outcome variable (here, infant mortality) in a treatment group (here, babies born in countries with a change—specifically, an increase—in paid maternity leave duration) with the average change over time in the outcome variable in a control group (here, babies born in countries without a change in paid maternity leave duration).

**What Did the Researchers Do and Find?:**

The researchers assembled a panel of about 300,000 live births in 20 LMICs between 2000 and 2008 using birth history data collected by the Demographic and Health Surveys (which collect information on the demographic, health, and other characteristics of a nationally representative sample of households). The researchers merged these observational data with information on the duration of paid maternity leave provided by each country and used the difference-in-differences approach to estimate the effect of an increase in paid maternity leave duration on the probability of infant (<1 year old), neonatal (<28 days old), and post-neonatal (between 28 days and 1 year old) mortality. Average rates of infant, neonatal, and post-neonatal mortality over the study period were 55.2, 30.7, and 23.0 per 1,000 live births, respectively. Each additional month of paid maternity leave was associated with 7.9 fewer infant deaths per 1,000 live births, a relative reduction in infant mortality of 13%. Notably, the reduction in infant mortality with increased duration of paid maternity leave was concentrated in the post-neonatal period.

**What Do These Findings Mean?:**

These findings suggest that policies that increase the duration of paid maternity leave could help to reduce infant mortality in the 20 LMICs included in this study. These findings may not be generalizable to all LMICs. Moreover, their accuracy may be limited by confounding. That is, unmeasured characteristics—rather than changes in paid maternity leave duration—could be responsible for the observed changes in infant mortality. Although the researchers adjusted for many possible confounders in their analysis, there may be some residual confounding from unmeasured time-varying confounders such as other policy changes made during the study period. Further work is now needed to determine the mechanisms that underlie the observed association between increased duration of paid maternity leave and reduction in infant mortality in LMICs. Paid maternity leave might, for example, improve infant health by giving new mothers time to breastfeed, care for ill babies, or ensure that their babies receive their childhood vaccinations. Finally, before LMICs introduce new policies on paid maternity leave, the optimal balance of paid leave from employment before and after delivery needs to be evaluated.

**Additional Information:**

This list of resources contains links that can be accessed when viewing the PDF on a device or via the online version of the article at http://dx.doi.org/10.1371/journal.pmed.1001985.

- The World Health Organization (WHO) provides information on child health and child mortality, and on global efforts to reduce child mortality (available in several languages); its 2009 publication *Home Visits for the Newborn child: A Strategy to Improve Survival* is available
- The United Nations Children’s Fund (UNICEF) works for children’s rights, survival, development, and protection around the world; it provides information about the failure to meet MDG4 and a link to a 2015 report on global levels and trends in child mortality; its UNICEF data website provides further detailed statistics about child health and mortality
- The *Millennium Development Goals 2015 Report* is available
- The Healthy Newborn Network is an online community of more than 80 partner organizations that addresses critical knowledge gaps in newborn health
- Wikipedia has pages on paid parental leave around the world, quasi-experiments, and the difference-in-differences analytical approach (note that Wikipedia is a free online encyclopedia that anyone can edit; available in several languages)

## Introduction

Paid leave for new parents, often specifically designated for new mothers, is a standard social benefit in most of the world. Over 180 countries have enacted legislation granting paid leave from employment in connection with the birth of a child, either in the form of maternity leave or gender-neutral parental leave [1].

Paid maternity and parental leave policies are consistently associated with improvements in child health in high-income countries [1–5]. For example, Ruhm found that increases in weeks of paid leave were associated with lower infant mortality in 16 European countries, although effects on perinatal and neonatal mortality were more modest [3]. Similarly, Tanaka showed that increases in paid parental leave were associated with decreases in perinatal, neonatal, post-neonatal, infant, and child mortality in a sample of 18 Organisation for Economic Co-operation and Development countries [4]. Recent work also showed that unpaid maternal leave provided through the Family and Medical Leave Act of 1993 in the United States was associated with decreases in neonatal, post-neonatal, and infant mortality, but only among women who were married and had graduated from college, suggesting that women of lower socioeconomic position may have been unable to take unpaid leave [5].

Paid maternity leave may affect neonatal and infant mortality through several mechanisms. First, paid maternity leave may increase access to pre- and postnatal health services. Some maternity leave policies allow for a short period of leave to be taken immediately prior to birth, which might increase receipt of third-trimester prenatal care. In the postnatal period, mothers able to take leave from employment may have more time to care for an ill child and seek medical care when necessary. Second, policies that provide income and ensure job protection during maternity leave may benefit women economically, reduce stress in the prenatal period, and improve maternal health after birth [6–8]; these factors might reduce adverse birth outcomes, including preterm birth and low birth weight [9–11]. Third, paid maternity leave might facilitate preventive care; for example, women able to take leave from employment are more likely to initiate breastfeeding and to continue breastfeeding for longer durations [12–15]. Paid maternity leave may also improve adherence to childhood vaccination schedules [16–18]. These mechanisms may interact synergistically; for example, increased income may be associated with more access to resources to support healthy behaviors and child-rearing practices [19,20].

Extant evaluations of paid maternity leave policies have been conducted almost exclusively in high-income countries. Whether these results can be generalized to lower-income contexts is unclear, given differences in the nature of non-parental child-care options as well as in women’s participation in the formal economy. In this study, we provide the first evaluation, to our knowledge, of whether paid maternity leave policies affect infant (<1 y), neonatal (<28 d), and post-neonatal (between 28 d and 1 y after birth) mortality in low- and middle-income countries (LMICs). We constructed a database of maternity leave policies for LMICs over time, merged this information to a sample of live births occurring between 2000 and 2008 in 20 LMICs surveyed as part of the Demographic and Health Surveys (DHS), and utilized a difference-in-differences approach to estimate the influence of paid maternity leave on mortality during the first year of life.

## Methods

### Sample

Information on birth outcomes was derived from the DHS. The authors had full access to the DHS data from which the analytic sample is derived. The DHS collect comparable information on demographic, socioeconomic, nutritional, behavioral, fertility, and health characteristics from a nationally representative sample of households in LMICs using a two-stage cluster sampling design. Individuals are selected for interviews from household rosters; information is collected on women of reproductive age (15–49 y), men (usually aged 15–54 or 15–59 y), and children under the age of 5 y. Trained interviewers and standardized tools and measurement techniques are used to ensure comparability of surveys across countries and survey waves. Further details regarding sampling strategies and study procedures are available elsewhere [21,22].

Our sample comprised all live births occurring to DHS respondents from 20 LMICs between 2000 and 2008. These 20 countries were selected because they administered at least two DHS surveys between 2001 and 2011, which permitted analyses of policy changes occurring within countries over time. Briefly, mothers surveyed in the DHS were asked to provide information concerning all children born alive in the previous 5 y. These data were used to construct a panel of live births, each with information on vital status, over a consistent set of years and countries. We created two separate samples, one for our analyses of infant and post-neonatal mortality and the other for analyses of neonatal mortality. We restricted these samples to the 282,836 and 304,294 live births that occurred at least 1 y and at least 28 d prior to the DHS interview date, respectively, in order to ascertain whether each child survived the infant (1 y) and neonatal (28 d) periods following birth. After further excluding observations with missing information on key covariates, our samples were composed of 282,751 live births between 2000 and 2007 for analyses of infant and post-neonatal mortality and 304,201 live births between 2001 and 2008 for analyses of neonatal mortality. Table 1 reports survey years and sample sizes for the sampled countries. The Institutional Review Board of McGill University reviewed and approved this study.

**Table 1:** Demographic and Health Surveys survey years, sample sizes, and rates of infant, neonatal, and post-neonatal mortality.

Country	DHS Survey Years	Infant Sample		Neonatal Sample		Post-Neonatal Sample	
		Number of Births	Weighted Percent Death	Number of Births	Weighted Percent Death	Number of Births	Weighted Percent Death
**Treated countries** ^a^							
Bangladesh	2004, 2007, 2011	11,739	5.98%	13,943	3.68%	11,739	1.77%
Kenya	2003, 2008	9,547	6.04%	9,015	3.18%	9,547	2.98%
Lesotho	2004, 2009	5,774	8.74%	6,696	4.23%	5,774	4.46%
Uganda	2006, 2011	13,093	6.79%	15,330	2.90%	13,093	3.83%
Zimbabwe	2005, 2010	8,035	5.54%	8,955	2.40%	8,035	3.10%
All treated countries		48,188	6.08%	53,939	3.50%	48,188	2.20%
**Control countries** ^b^							
Armenia	2005, 2010	2,211	2.15%	2,450	1.36%	2,211	0.77%
Bolivia	2003, 2008	13,539	5.03%	14,742	2.66%	13,539	2.44%
Colombia	2005, 2010	26,023	1.84%	26,607	1.18%	26,023	0.60%
Egypt	2005, 2008	18,376	2.81%	19,595	1.83%	18,376	1.04%
Ghana	2003, 2008	5,008	5.26%	5,460	3.34%	5,008	2.15%
Honduras	2005, 2011	17,319	2.51%	19,341	1.59%	17,319	0.89%
Cambodia	2005, 2010	13,352	6.38%	14,710	2.73%	13,352	3.28%
Madagascar	2003, 2008	15,452	5.22%	15,715	2.75%	15,452	2.60%
Malawi	2004, 2010	25,165	6.89%	28,857	2.88%	25,165	4.00%
Nigeria	2003, 2008	32,683	7.98%	35,137	4.12%	32,683	3.87%
Nepal	2006, 2011	9,342	4.59%	9,506	3.46%	9,342	1.33%
Philippines	2003, 2008	10,622	2.86%	11,064	1.68%	10,622	1.05%
Rwanda	2005, 2010	14,329	7.06%	15,130	3.11%	14,329	3.82%
Senegal	2005, 2010	17,958	5.69%	18,845	3.10%	17,958	2.25%
Tanzania	2004, 2010	13,184	5.96%	13,103	2.70%	13,184	3.03%
All control countries		234,563	5.16%	250,262	2.78%	234,563	2.36%
**Total**		282,751	5.52%	304,201	3.07%	282,751	2.30%

^a^Treated countries are countries that experienced a change in the duration of any paid leave.

^b^Control countries are countries that did not experience a change in the duration of any paid leave.

### Measures

The exposure of interest in our study was the legislated length of paid maternity leave for each country and year. Data on current maternity leave policies for each sampled country were provided by the University of California Los Angeles World Legal Rights Data Centre, and data on past maternity leave policies back to 1995 were collected by McGill University’s Maternal and Child Health Equity (MACHEquity) research program. We defined the duration of paid maternity leave as the legislated length of paid leave available to mothers only. We did not distinguish leave that could be taken in the prenatal period from leave that could be taken after birth; however, few maternity leave policies in LMICs mandate specifically prenatal versus postnatal leave. Further details regarding the collection and coding of global maternity leave policies are available elsewhere [1].

The outcome variables—infant, neonatal, and post-neonatal mortality—were measured using the 5-y birth histories provided by women interviewed in the DHS. We created binary indicators for infant, neonatal, and post-neonatal mortality to measure whether each child died within 1 y, within the first 28 d of life, or between 28 d and 1 y after birth, respectively.

We collected information on potential confounders and other characteristics based on a review of the literature on determinants of neonatal and infant mortality in LMICs [23–31]. Individual and household-level socio-demographic characteristics included the child’s gender, mother’s educational attainment in completed years, urban/rural residence, and household socioeconomic status (SES). Household SES, categorized into five equal groups using quintiles, was determined from the continuous wealth index provided by the DHS, which is based on ownership of specific assets (e.g., bicycle, radio, and television), environmental conditions, and housing characteristics (e.g., type of water source, sanitation facilities, materials used for housing construction), and was constructed using the method proposed by Filmer and Pritchett [32,33]. Birth characteristics included the interval between births (defined as short if it was less than 24 mo), maternal age at the time of each birth (categorized as <20, 20–39, or 40 y and older), and whether there was a skilled birth attendant present at the time of delivery. Aside from the length of paid leave, another defining feature of paid maternity leave policies is the percentage at which wages are replaced. Because changes in the wage replacement rate sometimes coincided (i.e., in Lesotho) with changes in the legislated length of leave, we controlled for the wage replacement rate as a potential confounder. Additional time-varying country-level covariates included levels of economic development (measured by gross domestic product [GDP] per capita based on purchasing power parity [PPP], in constant 2005 international dollars), female labor force participation (the proportion of women age 15 y and older in the labor force), per capita total health expenditure (PPP, in constant 2005 international dollars), and per capita government health expenditure (PPP, in constant 2005 international dollars), which were available from the World Bank’s World Development Indicators and Global Development Finance databases [34].

### Statistical Analysis

We linked data on paid maternity leave policies to outcomes and covariates from the DHS by country and birth year and examined the effects of an increase in the duration of paid maternity leave on the risk of mortality during the first year of life. Specifically, we estimated for birth *i* the effect of an additional month of paid leave (lagged by 1 y to respect the temporal ordering between the policy and outcome) on the probabilities of infant, neonatal, and post-neonatal mortality using a linear probability model of the general form *Y*_*ijt*_ = α_*j*_ + β*M*_*jt*−1_ + λ_*t*_ + ϵ_*ijt*_, where β measures the effect of a 1-mo increase in maternity leave in the prior year, *M*_*jt*−1_ (where *j* indexes the country and *t* the birth year), on infant, neonatal, and post-neonatal death, *Y*_*ijt*_. We included fixed effects for country (α_*j*_) and year (λ_*t*_) to control for unobserved time-invariant confounders that vary across countries and any temporal trends in mortality that are shared across countries, respectively. The effects of paid maternity leave policies were therefore identified by changes in outcomes occurring within countries that modified their maternity leave policies during the study period (treated countries) relative to corresponding changes in outcomes in countries that did not modify their policies during the study period (control countries).

In the first model, we estimated the effect of an additional month of paid maternity leave on infant, neonatal, and post-neonatal mortality after including country and year fixed effects (Model 1). In the second model, we additionally controlled for measured individual, household, and country-level characteristics, including the lagged wage replacement rate, natural log per capita GDP, and female labor force participation (Model 2). In the third model, we included controls for natural log per capita total and government health expenditures (Model 3); these data were unavailable for all years for Zimbabwe, and observations from Zimbabwe were therefore dropped from Model 3 analyses. In order to examine potential nonlinearity in the effect of paid maternity leave, we introduced a quadratic duration-of-paid-leave variable into our fully adjusted model (Model 3). We also estimated the fully adjusted model on the risk ratio (RR) scale. All analyses incorporated respondent-level sampling weights to account for individual survey sample designs. Per DHS guidelines, we used information on the number of women aged 15–49 y in each survey-year, provided by the Population Division of the United Nations [35], and applied the de-normalization of standard weights approach described in the DHS *Sampling and Household Listing Manual* [36], in order to calculate an appropriate weight for each observation in the analyses. All models incorporated robust standard errors to account for clustering at the country level [37].

### Sensitivity Analyses

We conducted sensitivity analyses to assess the robustness of our main findings. First, because there is no standard metric for maternity leave policies, we measured the effects of paid leave in full-time equivalent (FTE) units, obtained by multiplying the legislated length of leave by the wage replacement rate, in addition to the duration of any paid leave. Further details regarding the calculation of FTE units of paid leave are available elsewhere [1,16]. Second, we examined whether results were sensitive to the inclusion of sampling weights. Third, we added to our primary exposure, paid maternity leave in year *t −* 1, parameters representing paid maternity leave in preceding years (*t −* 3, *t −* 2), the birth year (*t*), and subsequent years (*t* + 1, *t* + 2, *t* + 3). These analyses assessed if there were persistent effects of the policy changes, as well as whether observed effects were responses to policy changes that occurred in the period during or before the measurement of our outcomes, as we would expect if the effects of paid maternity leave policies were causal. Fourth, as described in S1 Text, we assessed whether our findings were sensitive to the particular selection of control countries. In these analyses, we examined trends in infant, neonatal, and post-neonatal mortality occurring in the decade before our treated countries changed their legislated length of paid maternity leave and then restricted the sample of controls to those countries with parallel pre-intervention trends.

## Results

Table 1 shows the rates of infant, neonatal, and post-neonatal mortality for the 20 DHS countries; average rates of infant, neonatal, and post-neonatal mortality over the study period were 55.2, 30.7, and 23.0 per 1,000 live births, respectively. Table 2 shows trends in paid maternity leave benefits and country-level characteristics for the five treated and 15 control countries; baseline values for key covariates are provided for each country in S1 Table and trends in the duration of paid leave for individual treated countries are shown in S1 Fig. For treated countries, the average length of paid leave increased from 7.6 (standard deviation [SD] = 5.4) wk in 2000 to 12.2 (SD = 3.8) wk in 2008. The average length of paid leave in control countries was 12.2 (SD = 3.0) wk.

**Table 2:** Trends in paid maternity leave benefits and country-level characteristics for treated and control countries, 2000–2008.

Characteristic	Exposure	2000	2001	2002	2003	2004	2005	2006	2007	2008
**Duration of any paid leave (weeks)**	Treated	7.6 (5.4)	7.6 (5.4)	7.6 (5.4)	7.6 (5.4)	7.6 (5.4)	8.0 (4.7)	10.5 (5.5)	11.3 (4.0)	12.2 (3.8)
	Control	12.2 (3.0)	12.2 (3,0)	12.2 (3.0)	12.2 (3.0)	12.2 (3.0)	12.2 (3.0)	12.2 (3.0)	12.2 (3.0)	12.2 (3.0)
**Wage replacement rate (percent)**	Treated	75.0 (43.3)	75.0 (43.3)	75.0 (43.3)	80.0 (44.7)	80.0 (44.7)	100.0 (0.0)	100.0 (0.0)	100.0 (0.0)	100.0 (0.0)
	Control	87.5 (21.1)	87.5 (21.1)	87.5 (21.1)	87.5 (21.1)	90.8 (18.6)	90.8 (18.6)	90.8 (18.6)	90.8 (18.6)	90.8 (18.6)
**GDP per capita based on PPP (2005 international dollars)**	Treated	486.8 (177.3)	496.6 (179.8)	486.3 (158.3)	474.8 (147.1)	478.7 (145.1)	484.4 (147.3)	498.8 (151.1)	513.7 (160.0)	513.1 (183.3)
	Control	790.2 (727.1)	802.6 (727.7)	816.0 (738.5)	847.7 (758.1)	886.9 (789.4)	927.0 (821.5)	975.0 (873.8)	1,031.1 (933.4)	1,070.8 (959.6)
**Per capita total health expenditure based on PPP (2005 international dollars)**	Treated	52.2 (18.7)	53.8 (18.6)	53.6 (18.5)	55.7 (19.7)	60.7 (22.6)	65.9 (22.4)	76.1 (27.1)	85.7 (34.5)	89.9 (39.2)
	Control	99.5 (91.1)	105.2 (95.8)	107.6 (98.4)	120.6 (100.8)	126.5 (98.9)	138.3 (110.3)	149.0 (123.8)	161.0 (142.4)	172.2 (147.9)
**Per capita government health expenditure based on PPP (2005 international dollars)**	Treated	23.6 (12.6)	23.4 (13.9)	18.5 (15.2)	19.6 (16.8)	19.5 (16.6)	19.7 (14.7)	23.4 (19.7)	28.4 (29.1)	30.7 (34.0)
	Control	51.0 (70.0)	54.4 (71.4)	55.9 (73.1)	62.6 (78.8)	63.3 (68.3)	70.4 (75.1)	78.0 (84.9)	82.8 (91.1)	89.1 (99.1)
**Female labor force participation (proportion of women in the labor force)**	Treated	54.3 (9.3)	54.8 (10.2)	55.3 (12.0)	55.3 (13.7)	55.2 (15.7)	54.0 (16.0)	53.5 (16.3)	53.0 (16.6)	52.5 (17.0)
** **	Control	54.3 (19.6)	54.3 (19.6)	54.2 (19.6)	54.3 (19.5)	54.0 (19.8)	53.5 (20.1)	52.5 (20.6)	51.7 (20.8)	51.6 (20.8)

Data are given as mean (SD). Treated countries are the five countries (Bangladesh, Kenya, Lesotho, Uganda, and Zimbabwe) that experienced a change in the duration of any paid leave during the study period. Control countries are the 15 countries that did not experience a change in the duration of any paid leave during the study period.

Tables 3, 4 and 5 show the effects of an additional month of paid maternity leave on the probability of infant, neonatal, and post-neonatal death, respectively. In the fully adjusted model (Model 3), an additional month of paid maternity leave was associated with 7.9 (95% CI 3.7, 12.0) fewer infant deaths per 1,000 live births. Each additional month of paid leave was associated with 2.9 (95% CI –0.2, 6.0) fewer neonatal and 4.4 (95% CI 0.9, 8.0) fewer post-neonatal deaths per 1,000 live births. On the RR scale, each additional month of paid leave was associated with a 13% (RR = 0.87, 95% CI 0.81, 0.93), 9% (RR = 0.91, 95% CI 0.83, 1.00), and 18% (RR = 0.82, 95% CI 0.64, 1.05) reduction in infant, neonatal, and post-neonatal mortality, respectively (S2 Table). There was some evidence for a nonlinear effect of an additional month of paid maternity leave on the probabilities of infant and post-neonatal mortality, but not neonatal mortality (S3 Table). In particular, an additional month of paid leave was associated with a larger absolute reduction in infant and post-neonatal mortality when shorter durations of paid leave were available (S2 Fig).

**Table 3:** Effect of a 1-mo increase in paid maternity leave on the number of infant deaths per 1,000 live births, Demographic and Health Surveys, 2000–2007.

Exposure	Model 1^a^ (*n* = 282,751)			Model 2^b^ (*n* = 282,751)			Model 3^c^ (*n* = 274,716)		
	Estimate	LCL	UCL	Estimate	LCL	UCL	Estimate	LCL	UCL
**Additional month of paid leave**	−6.2	−10.4	−2.0	−5.9	−11.0	−0.8	−7.9	−12.0	−3.7
**Individual and household-level covariates** ^d^									
Male gender				9.8	7.4	12.3	9.8	7.3	12.3
Mother’s education (years)				−1.5	−2.9	−0.1	−1.5	−3.0	−0.1
Household SES 2nd quintile				−4.1	−11.0	2.9	−4.4	−11.3	2.4
Household SES 3rd wealth quintile				−1.9	−9.1	5.3	−2.2	−9.6	5.2
Household SES 4th wealth quintile				−9.2	−12.7	−5.6	−9.4	−13.0	−5.8
Household SES 5th quintile (highest)				−13.2	−19.6	−6.9	−13.6	−20.0	−7.1
Urban residence				0.4	−5.1	5.9	0.3	−5.3	6.0
Short birth interval (<24 mo)				33.1	23.1	43.1	32.9	22.7	43.1
Maternal age 20–39 y				−23.8	−30.9	−16.8	−24.0	−31.0	−16.9
Maternal age ≥40 y				−6.6	−17.2	4.1	−6.5	−17.2	4.2
Skilled attendant at delivery				−1.7	−9.9	6.5	−1.1	−9.3	7.1
**Country-level covariates**									
Wage replacement rate				0.0	−0.1	0.2	0.1	−0.1	0.3
ln GDP per capita				−30.9	−75.6	13.9	−10.2	−93.7	73.3
Female labor force participation				0.2	−0.7	1.1	0.6	−0.4	1.6
ln government health expenditure per capita							−7.7	−17.0	1.6
ln total health expenditure per capita							−13.9	−35.5	7.6

^a^Model 1 includes country and year fixed effects.

^b^Model 2 additionally controlled for measured individual, household, and country-level characteristics.

^c^Model 3 additionally controlled for per capita total and government health expenditures; these data were unavailable for all years for Zimbabwe, and observations from Zimbabwe were therefore dropped from Model 3.

^d^Reference categories for categorical variables are female gender, the first (lowest) household SES quintile, rural residence, birth interval ≥ 24 mo, maternal age < 20 y, and absence of a skilled attendant at delivery.

LCL, lower confidence limit of 95% CI; ln, natural log; UCL, upper confidence limit of 95% CI.

**Table 4:** Effects of a 1-mo increase in paid maternity leave on the number of neonatal deaths per 1,000 live births, Demographic and Health Surveys, 2001–2008.

Exposure	Model 1^a^ (*n* = 304,201)			Model 2^b^ (*n* = 304,201)			Model 3^c^ (*n* = 295,246)		
	Estimate	LCL	UCL	Estimate	LCL	UCL	Estimate	LCL	UCL
**Additional month of paid leave**	−3.3	−5.1	−1.6	−2.6	−5.0	−0.2	−2.9	−6.0	0.2
**Individual and household-level covariates** ^d^									
Male gender				8.3	6.9	9.8	8.4	6.8	9.9
Mother’s education (years)				−0.5	−1.3	0.2	−0.5	−1.3	0.2
Household SES 2nd quintile				−0.3	−4.2	3.6	−0.4	−4.4	3.5
Household SES 3rd wealth quintile				0.1	−3.2	3.3	−0.1	−3.4	3.2
Household SES 4th wealth quintile				−2.2	−6.1	1.8	−2.3	−6.3	1.6
Household SES 5th quintile (highest)				−4.5	−9.8	0.9	−4.7	−10.1	0.8
Urban residence				−1.5	−5.0	2.0	−1.6	−5.1	2.0
Short birth interval (<24 mo)				14.2	10.5	17.9	14.2	10.4	18.0
Maternal age 20–39 y				−15.9	−21.6	−10.1	−15.9	−21.7	−10.1
Maternal age ≥40 y				−7.2	−20.4	5.9	−7.3	−20.6	5.9
Skilled attendant at delivery				4.8	−2.9	12.5	5.0	−2.7	12.8
**Country-level covariates**									
Wage replacement rate				−0.1	−0.3	0.0	−0.1	−0.3	0.2
ln GDP per capita				−22.1	−48.7	4.5	−34.4	−93.5	24.7
Female labor force participation				0.1	−0.6	0.9	0.5	−0.7	1.8
ln government health expenditure per capita							−5.6	−14.7	3.5
ln total health expenditure per capita							1.0	−9.5	11.5

^a^Model 1 includes country and year fixed effects.

^b^Model 2 additionally controlled for measured individual, household, and country-level characteristics.

^c^Model 3 additionally controlled for per capita total and government health expenditures; these data were unavailable for all years for Zimbabwe, and observations from Zimbabwe were therefore dropped from Model 3.

^d^Reference categories for categorical variables are female gender, the first (lowest) household SES quintile, rural residence, birth interval ≥ 24 mo, maternal age < 20 y, and absence of a skilled attendant at delivery.

LCL, lower confidence limit of 95% CI; ln, natural log; UCL, upper confidence limit of 95% CI.

**Table 5:** Effects of a 1-mo increase in paid maternity leave on the number of post-neonatal deaths per 1,000 live births, Demographic and Health Surveys, 2000–2007.

Exposure	Model 1^a^ (*n* = 282,751)			Model 2^b^ (*n* = 282,751)			Model 3^c^ (*n* = 274,716)		
	Estimate	LCL	UCL	Estimate	LCL	UCL	Estimate	LCL	UCL
**Additional month of paid leave**	−3.6	−6.8	−0.4	−3.5	−7.3	0.3	−4.4	−8.0	−0.9
**Individual and household-level covariates** ^d^									
Male gender				1.4	−0.4	3.2	1.3	−0.5	3.1
Mother’s education (years)				−1.0	−1.6	−0.5	−1.0	−1.6	−0.5
Household SES 2nd quintile				−1.1	−4.3	2.1	−1.3	−4.5	2.0
Household SES 3rd wealth quintile				−2.4	−4.8	0.0	−2.4	−4.9	0.1
Household SES 4th wealth quintile				−4.6	−7.6	−1.7	−4.6	−7.7	−1.6
Household SES 5th quintile (highest)				−7.3	−11.3	−3.3	−7.3	−11.4	−3.2
Urban residence				1.7	−1.4	4.8	1.7	−1.4	4.8
Short birth interval (<24 mo)				15.9	11.0	20.7	15.7	10.8	20.7
Maternal age 20–39 y				−6.8	−11.1	−2.5	−6.8	−11.2	−2.5
Maternal age ≥40 y				−0.8	−7.4	5.7	−0.7	−7.3	5.9
Skilled attendant at delivery				−3.5	−5.3	−1.8	−3.5	−5.3	−1.6
**Country-level covariates**									
Wage replacement rate				0.0	−0.1	0.2	0.1	−0.1	0.2
ln GDP per capita				−11.1	−45.6	23.4	9.9	−53.3	73.2
Female labor force participation				−0.1	−0.8	0.7	0.0	−0.7	0.8
ln government health expenditure per capita							−3.3	−12.4	5.8
ln total health expenditure per capita							−8.4	−21.7	4.8

^a^Model 1 includes country and year fixed effects.

^b^Model 2 additionally controlled for measured individual, household, and country-level characteristics.

^c^Model 3 additionally controlled for per capita total and government health expenditures; these data were unavailable for all years for Zimbabwe, and observations from Zimbabwe were therefore dropped from Model 3.

^d^Reference categories for categorical variables are female gender, the first (lowest) household SES quintile, rural residence, birth interval ≥ 24 mo, maternal age < 20 y, and absence of a skilled attendant at delivery.

LCL, lower confidence limit of 95% CI; ln, natural log; UCL, upper confidence limit of 95% CI.

There were pronounced socioeconomic gradients in infant and post-neonatal, but not neonatal, mortality; there were, on average, 13.6 (95% CI 7.1, 20.0) fewer infant deaths per 1,000 live births in households in the highest quintile of household SES compared to the lowest quintile. Birth characteristics, including short birth interval (<24 mo) and lower maternal age (<20 y), were consistently associated with neonatal and infant mortality. For example, a short birth interval was associated with an additional 32.9 (95% CI 22.7, 43.1) infant, 14.2 (95% CI 10.4, 18.0) neonatal, and 15.7 (95% CI 10.8, 20.7) post-neonatal deaths per 1,000 live births. Country-level characteristics were not associated with mortality.

### Sensitivity Analyses

Results from sensitivity analyses for infant, neonatal, and post-neonatal outcomes are shown in S4 Table, S5 Table, and S6 Table, respectively. The effects of paid leave policies on infant mortality were fairly robust. When paid leave was measured in FTE units, an additional month of leave was associated with a smaller decrease in infant mortality of 3.6 (95% CI −2.4, 9.6) fewer deaths per 1,000 live births (Model A). Unweighted estimates suggested that an additional month of paid leave was associated with 10.3 (95% CI 4.9, 15.7) fewer infant deaths per 1,000 live births (Model B). The effects of paid leave on infant mortality were isolated to responses to policy changes that occurred in the year preceding the measurement of the outcome (Model C). Additionally, our results were qualitatively similar after we restricted our sample of control countries to those with similar pre-intervention trends in infant mortality between 1990 and 2000 (Model D). By contrast, the effects of paid maternity leave policies on neonatal mortality varied substantially across alternative model specifications. In particular, an increase in the duration of paid leave was no longer associated with neonatal mortality in models that were unweighted (Model B). Additionally, estimates of the effects of paid leave modeled separately in years before and after the policy change were relatively imprecise (Model C). The effect of paid maternity leave on post-neonatal mortality was similar in unweighted and weighted models (Model B); however, the effects of lagged and lead effects were unstable and difficult to interpret (Model C).

## Discussion

We merged longitudinal information on the legislated duration of paid maternity leave in 20 LMICs with a panel of approximately 300,000 live births recorded in DHS surveys in those countries between 2000 and 2008 and used this dataset to conduct the first evaluation, to our knowledge, of the impact of paid maternity leave on infant mortality in LMICs. Difference-in-differences analyses suggested that each additional month of paid maternal leave was associated with approximately eight fewer infant deaths per 1,000 live births. These findings were relatively robust to alternative model specifications.

Policies guaranteeing new mothers paid leave from work provide new mothers with the opportunity to rest and recover after childbirth, increase their job protection and labor force attachment [38], and may benefit their mental and physical health [7,8,39]. Moreover, a growing literature suggests that paid maternity leave policies have a beneficial effect on maternal health behaviors, including breastfeeding [12–15], and infant health in higher-income countries [3–5]. Given the lack of evidence, it has been unclear if these findings can be generalized to poorer countries, where rates of female labor force participation in the formal economy are generally lower. Our results indicate that more generous paid maternity leave policies may have an even greater potential to reduce infant mortality in LMICs than in higher-income countries [3,4], both in absolute and relative terms, although prior work in high-income countries considered parental leave rather than maternity leave per se. Furthermore, our findings suggest that the benefits of additional paid maternity leave in terms of reducing infant mortality were larger when shorter durations of paid leave were available. Heterogeneity in the impact of paid maternity leave across countries warrants further investigation. Although a smaller proportion of women may be eligible for leave in LMICs than in high-income countries, and leave policy implementation may be poorer, the actual benefit to child health conditional on being eligible may be substantial, given higher infant mortality rates in poorer contexts. Furthermore, a greater proportion of women in LMICs may be benefiting from leave benefits than we might anticipate. Labor reforms targeting the formal economy may have spillover effects that influence workers in the informal economy. Moreover, many international labor standards and national laws are constructed to encompass all types of workers.

Similar to observations from higher-income countries [3,4], extending the duration of paid leave available to new mothers resulted in larger reductions in mortality in the post-neonatal than in the neonatal period. An increase in the duration of paid leave available to mothers might influence postnatal factors, including the duration of breastfeeding and vaccination uptake, which are consistently associated with better infant health [40–42]. Recent work, for example, showed that increases in paid maternity leave were associated with increased uptake of diphtheria, pertussis, and tetanus immunizations [43]. Epidemiologic evidence suggests that neonatal mortality, by contrast, is determined to a greater extent by antenatal factors that are less likely to be influenced by increases in maternity leave, unless that leave is taken before birth; these factors include maternal and reproductive factors, such as maternal age and birth spacing, as well as health services characteristics, including the use of antenatal care and the place of delivery [44–50]. Further research into the effects of maternity leave policies on utilization of health services and behavioral risk factors for neonatal and infant mortality is needed.

Our findings suggest there is potential for improving infant health by increasing the duration of paid maternity leave in LMICs. Recently, the Countdown to 2015 report suggested that only one-third of the 75 “Countdown countries,” those accounting for more than 95% of global maternal and infant deaths, achieved the Millennium Development Goal 4 of reducing child (<5 y) mortality by two-thirds by 2015 [51]. As others have argued [52], in order to end preventable deaths among children, countries will need to address neonatal and infant deaths more effectively. Data from the MACHEquity research program showed that one-quarter of the Countdown countries provided less than 12 wk of paid maternity leave in 2012 (median = 12.9 wk). Our findings suggest that social interventions, in addition to health policy interventions [53], warrant further discussion in the post-2015 development agenda. More generous paid maternity leave policies represent a potential instrument for facilitating early-life interventions and reducing infant mortality in LMICs.

There were limitations to our study. First, as in any non-experimental study, there is the potential for unmeasured confounding. However, we controlled for potential confounding by individual, household, and country-level characteristics. We also included fixed effects for country and year to account for unobserved time-invariant confounders that vary across countries and any temporal trends in mortality that are shared across countries, respectively. Therefore, any unmeasured confounders that would remain to bias our estimates would have to coincide with policy changes occurring within treated countries and also influence mortality, which markedly reduces the list of potential unobserved confounders. However, the adoption of several policies, including paid leave policies, simultaneously would conflate effects and potentially bias our results. Second, the determination of our outcomes, neonatal and infant mortality, depends on maternal recall, and mothers may underreport the births and deaths of children who are not alive at the time of the interview. Such underreporting would bias our estimates only if it were different between our treatment and control countries. Third, our measure of paid maternity leave is calculated based on legislated maternity leave and does not account for other leave (i.e., parental leave) that might also be available to mothers. Fourth, in order to model the longitudinal effect of maternity leave policies on neonatal and infant mortality, we limited our analyses to 20 selected LMICs with at least two DHS surveys between 2001 and 2011; the inclusion of sampling weights allows us to generalize our results to these 20 countries, but not to all LMICs.

Caveats considered, our analyses suggest that an additional month of paid maternity leave is associated with as much as a 13% reduction in infant mortality in LMICs. From a policy planning perspective, further work is needed to elucidate the mechanisms that explain the beneficial effects of paid maternity leave on infant mortality and to evaluate the optimal balance of leave from employment prior to and following delivery, as well as varying levels of compensation. Further work is also needed to document the effects of paid maternity leave on women’s labor force participation, health, and well-being in LMICs.

## Supporting Information

S1 Fig Trends in the duration of paid maternity leave for individual treated and aggregated control countries, 2000–2008. (TIF)

S2 Fig Predicted probabilities, with 95% confidence intervals, of infant, neonatal, and post-neonatal death at different durations of paid maternity leave. Top: infant; middle: neonatal; bottom: post-neonatal.
(TIF)

S3 Fig Trends in infant, neonatal, and post-neonatal deaths per 1,000 live births among all treated and control countries. Top: infant; middle: neonatal; bottom: post-neonatal.
(TIF)

S4 Fig Trends in infant and neonatal deaths per 1,000 live births among treated and control countries, after restricting to control countries with similar trends. Top: infant; bottom: neonatal.
(TIF)

S1 Table Baseline values of key covariates for treated and control countries. (DOCX)

S2 Table Models examining the effect of paid maternity leave on infant, neonatal, and post-neonatal morality on the risk ratio scale, Demographic and Health Surveys. (DOCX)

S3 Table Models examining potential nonlinearity in the effect of paid maternity leave on infant, neonatal, and post-neonatal morality by introducing a quadratic duration-of-paid-leave variable to a fully adjusted model, Demographic and Health Surveys. (DOCX)

S4 Table Sensitivity analyses comparing the effects of an increase in paid maternity leave on the number of infant deaths per 1,000 live births across different model specifications, Demographic and Health Surveys, 2000–2007. (DOCX)

S5 Table Sensitivity analyses comparing the effects of an increase in paid maternity leave on the number of neonatal deaths per 1,000 live births across different model specifications, Demographic and Health Surveys, 2001–2008. (DOCX)

S6 Table Sensitivity analyses comparing the effects of an increase in paid maternity leave on the number of post-neonatal deaths per 1,000 live births across different model specifications, Demographic and Health Surveys, 2000–2007. (DOCX)

S1 Text RECORD statement. Checklist of items, extended from the STROBE statement, that should be reported in observational studies using routinely collected health data.
(DOCX)

S2 Text Examination of pre-intervention trends. (DOCX)

S3 Text Analysis history. (DOCX)

## Abbreviations

DHS: Demographic and Health Surveys
FTE: full-time equivalent
GDP: gross domestic product
LMICs: low- and middle-income countries
MACHEquity: Maternal and Child Health Equity
PPP: purchasing power parity
RR: risk ratio
SD: standard deviation
SES: socioeconomic status
