# Supplementary figures and images for: Increased Duration of Paid Maternity Leave Lowers Infant Mortality in Low- and Middle-Income Countries: A Quasi-Experimental Study
Source: PLoS Med. 2016 Mar 29;13(3):e1001985. doi: 10.1371/journal.pmed.1001985 (PMC4811564; doi:10.1371/journal.pmed.1001985)

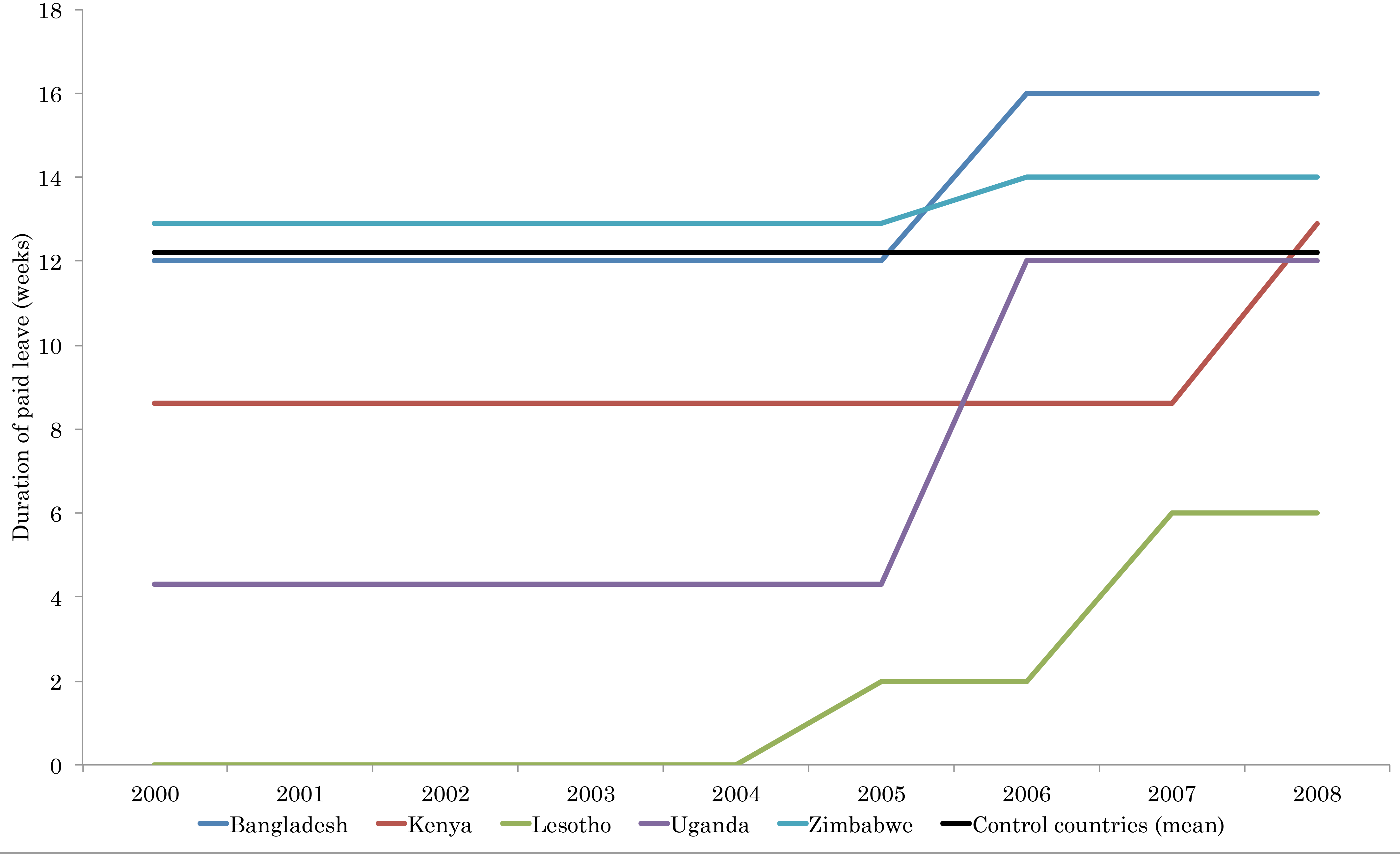

Supplement: S1 Fig — (TIF) [file pmed.1001985.s001.tif]

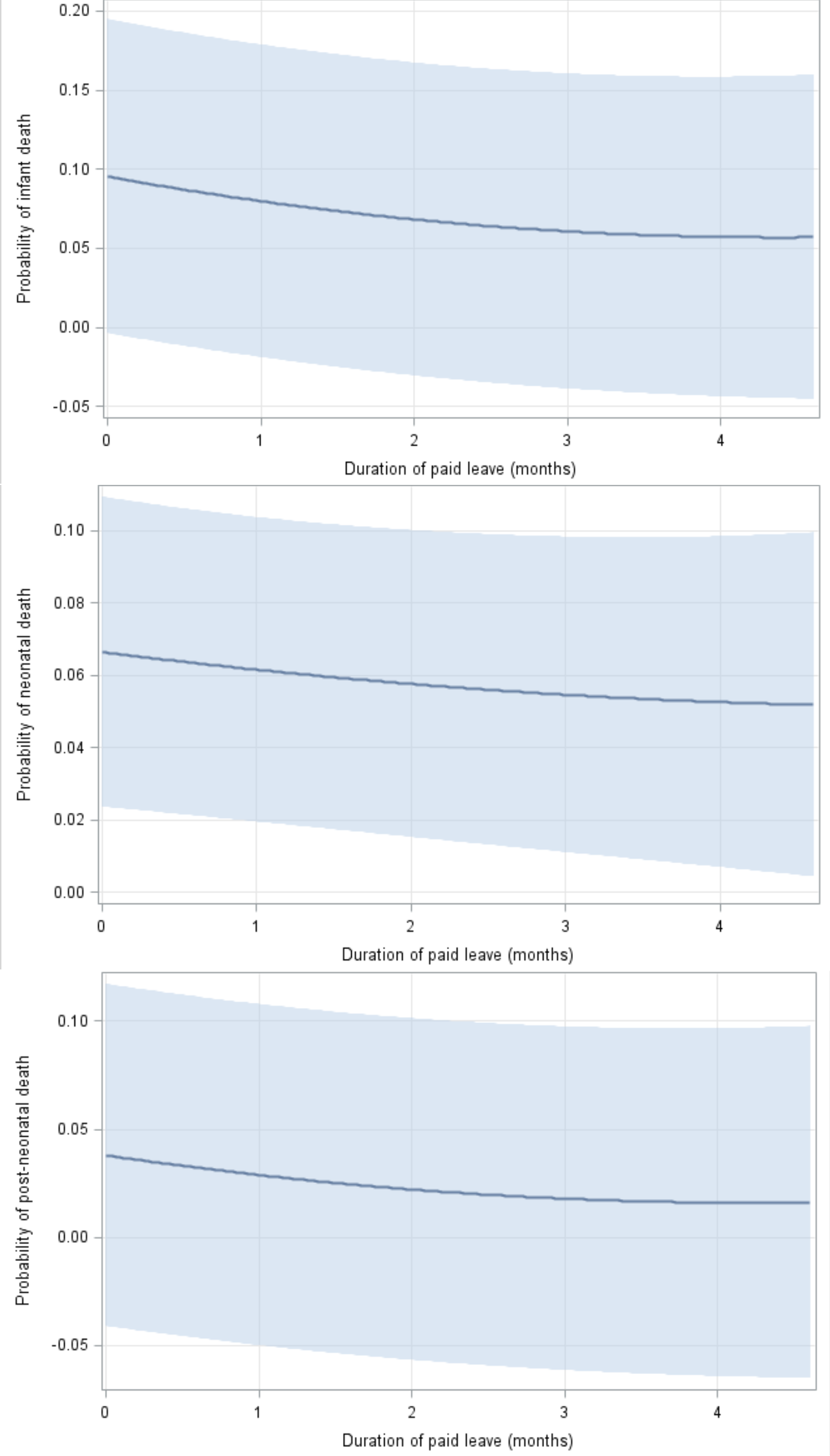

Supplement: S2 Fig — Top: infant; middle: neonatal; bottom: post-neonatal. (TIF) [file pmed.1001985.s002.tif]

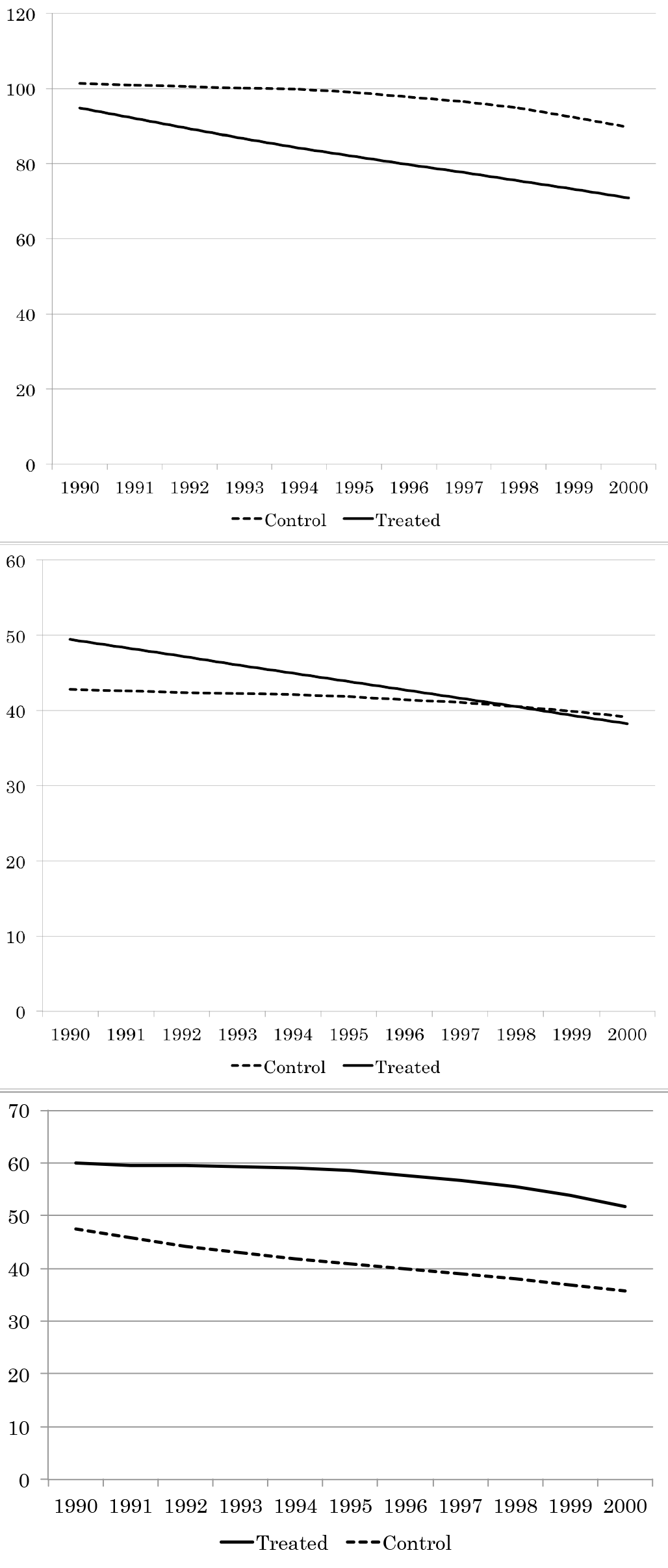

Supplement: S3 Fig — Top: infant; middle: neonatal; bottom: post-neonatal. (TIF) [file pmed.1001985.s003.tif]

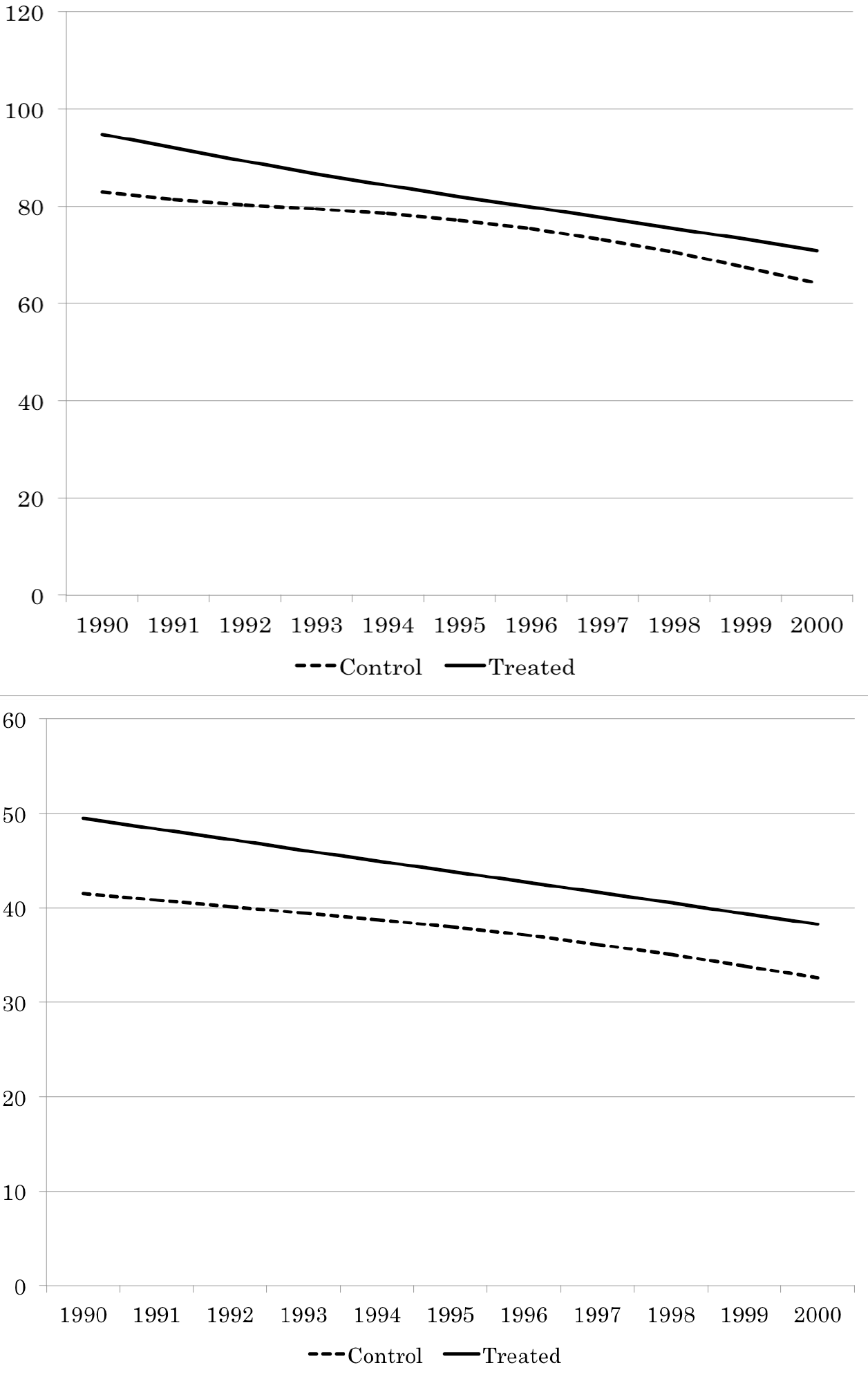

Supplement: S4 Fig — Top: infant; bottom: neonatal. (TIF) [file pmed.1001985.s004.tif]
