# Supplementary material for: Increased Duration of Paid Maternity Leave Lowers Infant Mortality in Low- and Middle-Income Countries: A Quasi-Experimental Study
Source: PLoS Med. 2016 Mar 29;13(3):e1001985. doi: 10.1371/journal.pmed.1001985 (PMC4811564; doi:10.1371/journal.pmed.1001985)
Supplement: S1 Table — (DOCX) [file pmed.1001985.s005.docx]

**Table S1.** Baseline values of key covariates for treated and control countries

| **Country** | **Duration of paid leave in weeks** | **Wage replacement rate** | **Weighted % infant death^1^** | **Weighted % neonatal death^1^** | **Weighted % post-neonatal death^1^** | **GDP per capita, PPP, 2005 international $** | **Female labor force participation** | **Per capita total health expenditure** | **Per capita government health expenditure** |
| --- | --- | --- | --- | --- | --- | --- | --- | --- | --- |
|  | *Treated countries* | | | | | | | | |
| Bangladesh | 12 | 100 | 7.37 | 4.98 | 2.38 | 349.5 | 51.8 | 24.2 | 9.4 |
| Kenya | 8.6 | 100 | 8.89 | 3.56 | 5.33 | 501.0 | 41.4 | 53.3 | 24.7 |
| Lesotho | 0 | 0 | 9.16 | 3.76 | 5.4 | 639.7 | 56.6 | 68.5 | 35.1 |
| Uganda | 4.3 | 100 | 8.7 | 4.03 | 4.68 | 268.5 | 67.3 | 45.6 | 12.2 |
| Zimbabwe | 12.9 | 75 | 5.96 | 2.89 | 3.07 | 675.6 | 54.6 | 69.5 | 36.7 |
| TREATED | 7.56 | 75 | 7.62 | 4.68 | 2.94 | 486.8 | 54.3 | 52.2 | 23.6 |
|  | *Control countries* | | | | | | | | |
| Armenia | 20 | 100 | 2.66 | 0.21 | 2.45 | 895.6 | 47.5 | 127.7 | 23.2 |
| Bolivia | 12.9 | 95 | 4.76 | 2.42 | 2.34 | 965.4 | 46.4 | 192.3 | 115.5 |
| Colombia | 12 | 100 | 1.87 | 0.97 | 0.9 | 3074.3 | 41.3 | 345.2 | 273.8 |
| Egypt | 12.9 | 100 | 2.29 | 1.08 | 1.21 | 1140.1 | 19.0 | 199.5 | 80.7 |
| Ghana | 12 | 50 | 4.99 | 3.25 | 1.73 | 445.9 | 54.4 | 44.9 | 21.8 |
| Honduras | 12 | 100 | 2.49 | 1.07 | 1.42 | 1241.0 | 37.3 | 169.8 | 92.1 |
| Cambodia | 12.9 | 50 | 9.12 | 4.31 | 4.81 | 329.4 | 71.7 | 58.1 | 11.9 |
| Madagascar | 14 | 100 | 7.05 | 3.76 | 3.29 | 286.0 | 74.3 | 29.8 | 19.8 |
| Malawi | 8 | 100 | 7.78 | 2.8 | 4.97 | 221.3 | 61.7 | 35.9 | 16.4 |
| Nigeria | 12 | 50 | 7.1 | 3.56 | 3.54 | 678.6 | 30.5 | 60.0 | 20.1 |
| Nepal | 7.4 | 100 | 5.59 | 3.81 | 1.78 | 296.8 | 79.7 | 43.3 | 10.6 |
| Philippines | 8.6 | 100 | 2.84 | 2 | 0.84 | 1060.6 | 37.6 | 76.8 | 36.5 |
| Rwanda | 12 | 67 | 8.44 | 4.19 | 4.26 | 210.7 | 77.1 | 24.7 | 9.7 |
| Senegal | 14 | 100 | 6.66 | 4.44 | 2.22 | 702.3 | 53.8 | 58.7 | 21.6 |
| Tanzania | 12 | 100 | 5.99 | 2.68 | 3.32 | 304.4 | 82.1 | 25.3 | 11.0 |
| CONTROL | 12.18 | 87.5 | 4.95 | 2.56 | 2.4 | 790.2 | 54.3 | 99.5 | 51.0 |
| TOTAL | 11 | 84.35 | 6.02 | 3.4 | 2.61 | 714.3 | 54.3 | 87.7 | 44.1 |

^1^ Estimates from 2001, the first year when data were available for all outcomes
