# Supplementary material for: Increased Duration of Paid Maternity Leave Lowers Infant Mortality in Low- and Middle-Income Countries: A Quasi-Experimental Study
Source: PLoS Med. 2016 Mar 29;13(3):e1001985. doi: 10.1371/journal.pmed.1001985 (PMC4811564; doi:10.1371/journal.pmed.1001985)
Supplement: S2 Table — (DOCX) [file pmed.1001985.s006.docx]

**Table S2.** Models examining the effect of paid maternity leave on infant, neonatal, and post-neonatal morality on the risk ratio scale, Demographic and Health Surveys

|  | **Infant mortality** | | | **Neonatal mortality** | | | **Post-neonatal mortality** | | |
| --- | --- | --- | --- | --- | --- | --- | --- | --- | --- |
|  | *RR^a^* | *LCL^b^* | *UCL* | *RR* | *LCL* | *UCL* | *RR* | *LCL* | *UCL* |
| **Additional month of paid leave** | **0.87** | **0.81** | **0.93** | **0.91** | **0.83** | **1.00** | **0.82** | **0.64** | **1.05** |
|  | *Individual and household-level covariates^c^* | | | | | | | | |
| Male gender | 1.19 | 1.15 | 1.24 | 1.32 | 1.23 | 1.40 | 1.06 | 0.98 | 1.15 |
| Mother's education (years) | 0.97 | 0.94 | 1.00 | 0.98 | 0.96 | 1.01 | 0.95 | 0.92 | 0.98 |
| 2nd wealth quintile | 0.94 | 0.85 | 1.03 | 0.99 | 0.90 | 1.09 | 0.96 | 0.86 | 1.07 |
| 3rd wealth quintile | 0.98 | 0.86 | 1.10 | 1.00 | 0.90 | 1.11 | 0.92 | 0.84 | 1.01 |
| 4th wealth quintile | 0.85 | 0.81 | 0.89 | 0.93 | 0.84 | 1.02 | 0.82 | 0.74 | 0.91 |
| 5th quintile (highest) | 0.76 | 0.67 | 0.86 | 0.85 | 0.72 | 0.99 | 0.67 | 0.57 | 0.78 |
| Urban residence | 1.00 | 0.91 | 1.09 | 0.94 | 0.85 | 1.04 | 1.08 | 0.93 | 1.26 |
| Short birth interval | 1.66 | 1.59 | 1.73 | 1.52 | 1.45 | 1.58 | 1.73 | 1.65 | 1.82 |
| Maternal age 20-39 | 0.68 | 0.64 | 0.71 | 0.64 | 0.59 | 0.69 | 0.75 | 0.70 | 0.80 |
| Maternal age >=40 | 0.89 | 0.76 | 1.04 | 0.83 | 0.62 | 1.12 | 0.92 | 0.80 | 1.07 |
| Skilled attendant at delivery | 0.99 | 0.85 | 1.14 | 1.20 | 0.97 | 1.49 | 0.85 | 0.75 | 0.96 |
|  | *Country-level covariates* | | | | | | | | |
| Wage replacement rate | 1.00 | 1.00 | 1.01 | 1.00 | 0.99 | 1.01 | 1.00 | 1.00 | 1.01 |
| ln GDP per capita | 1.03 | 0.31 | 3.41 | 0.31 | 0.05 | 1.95 | 1.32 | 0.33 | 5.32 |
| female labor force participation | 1.02 | 1.00 | 1.03 | 1.03 | 0.98 | 1.08 | 1.01 | 0.99 | 1.02 |
| ln government health expenditure | 0.87 | 0.76 | 1.00 | 0.79 | 0.59 | 1.05 | 0.93 | 0.69 | 1.25 |
| ln total health expenditure | 0.86 | 0.64 | 1.16 | 1.08 | 0.76 | 1.52 | 0.97 | 0.65 | 1.44 |
| Sample size | 274716 | | | 295246 | | | 274716 | | |

^a^ RR=risk ratio

^b^ LCL and UCL indicate lower and upper limits of the 95% confidence interval, respectively

^c^Reference categories for categorical variables are female (vs. male) gender, the 1^st^ (lowest) wealth quintile, rural (vs. urban) residence, longer (vs. <24 month) birth interval, lower (<20 year) maternal age, and absence (vs. presence) or a skilled attendant at delivery
