# Supplementary material for: Increased Duration of Paid Maternity Leave Lowers Infant Mortality in Low- and Middle-Income Countries: A Quasi-Experimental Study
Source: PLoS Med. 2016 Mar 29;13(3):e1001985. doi: 10.1371/journal.pmed.1001985 (PMC4811564; doi:10.1371/journal.pmed.1001985)
Supplement: S3 Table — (DOCX) [file pmed.1001985.s007.docx]

**Table S3.** Models examining potential non-linearity in the effect of paid maternity leave on infant, neonatal, and post-neonatal morality by introducing a quadratic duration of paid leave variable to a fully adjusted model, Demographic and Health Surveys

|  | **Infant mortality** | | | **Neonatal mortality** | | | **Post-neonatal mortality** | | |
| --- | --- | --- | --- | --- | --- | --- | --- | --- | --- |
|  | *Estimate* | *LCL^a^* | *UCL* | *Estimate* | *LCL* | *UCL* | *Estimate* | *LCL* | *UCL* |
| **Additional month of paid leave** | -17.6 | -25.4 | -9.8 | -5.4 | -11.1 | 0.4 | -10.4 | -15.8 | -4.9 |
| **Additional month of paid leave^2^** | 2.0 | 0.1 | 3.9 | 0.5 | -0.8 | 1.7 | 1.2 | -0.1 | 2.5 |
|  | *Individual and household-level covariates^b^* | | | | | | | | |
| Male gender | 9.8 | 7.3 | 12.3 | 8.4 | 6.8 | 9.9 | 1.3 | -0.5 | 3.1 |
| Mother's education (years) | -1.5 | -3.0 | -0.1 | -0.5 | -1.3 | 0.2 | -1.0 | -1.6 | -0.5 |
| 2nd wealth quintile | -4.4 | -11.3 | 2.4 | -0.4 | -4.4 | 3.5 | -1.3 | -4.5 | 2.0 |
| 3rd wealth quintile | -2.2 | -9.6 | 5.3 | -0.1 | -3.4 | 3.2 | -2.4 | -4.9 | 0.1 |
| 4th wealth quintile | -9.4 | -13.0 | -5.8 | -2.3 | -6.3 | 1.6 | -4.6 | -7.7 | -1.6 |
| 5th quintile (highest) | -13.6 | -20.0 | -7.1 | -4.7 | -10.1 | 0.8 | -7.3 | -11.4 | -3.2 |
| Urban residence | 0.3 | -5.3 | 6.0 | -1.6 | -5.1 | 2.0 | 1.7 | -1.5 | 4.8 |
| Short birth interval | 32.9 | 22.7 | 43.1 | 14.2 | 10.4 | 18.0 | 15.7 | 10.8 | 20.7 |
| Maternal age 20-39 | -24.0 | -31.0 | -16.9 | -15.9 | -21.7 | -10.1 | -6.8 | -11.2 | -2.5 |
| Maternal age >=40 | -6.5 | -17.2 | 4.2 | -7.3 | -20.6 | 5.9 | -0.7 | -7.3 | 5.9 |
| Skilled attendant at delivery | -1.0 | -9.3 | 7.2 | 5.0 | -2.7 | 12.8 | -3.5 | -5.3 | -1.6 |
|  | *Country-level covariates* | | | | | | | | |
| Wage replacement rate | 0.2 | 0.0 | 0.3 | -0.1 | -0.3 | 0.2 | 0.1 | 0.0 | 0.2 |
| ln GDP per capita | -14.6 | -98.1 | 68.9 | -35.1 | -95.5 | 25.3 | 7.2 | -55.4 | 69.9 |
| female labor force participation | 0.6 | -0.4 | 1.6 | 0.5 | -0.8 | 1.8 | 0.0 | -0.7 | 0.7 |
| ln government health expenditure | -7.7 | -17.5 | 2.1 | -5.6 | -14.7 | 3.5 | -3.3 | -12.4 | 5.9 |
| ln total health expenditure | -13.6 | -35.4 | 8.1 | 1.0 | -9.4 | 11.5 | -8.2 | -21.5 | 5.0 |
| Sample size | 274716 | | | 295246 | | | 274716 | | |

^a^ LCL and UCL indicate lower and upper limits of the 95% confidence interval, respectively

^b^ Reference categories for categorical variables are female (vs. male) gender, the 1^st^ (lowest) wealth quintile, rural (vs. urban) residence, longer (vs. <24 month) birth interval, lower (<20 year) maternal age, and absence (vs. presence) or a skilled attendant at delivery
