# Supplementary material for: Increased Duration of Paid Maternity Leave Lowers Infant Mortality in Low- and Middle-Income Countries: A Quasi-Experimental Study
Source: PLoS Med. 2016 Mar 29;13(3):e1001985. doi: 10.1371/journal.pmed.1001985 (PMC4811564; doi:10.1371/journal.pmed.1001985)
Supplement: S4 Table — (DOCX) [file pmed.1001985.s008.docx]

**Table S4.** Sensitivity analyses comparing the effects of an increase in paid maternity leave on the number of infant deaths per 1000 live births across different model specificiations, Demographic and Health Surveys, 2000-2007

|  | **(a) FTE measure of maternity leave^a^** | | | **(b) Unweighted estimates^b^** | | | **(c) Analysis of lags and leads^c^** | | | **(d) Restricted set of control countries^d^** | | |
| --- | --- | --- | --- | --- | --- | --- | --- | --- | --- | --- | --- | --- |
|  | *Estimate* | *LCL^e^* | *UCL* | *Estimate* | *LCL* | *UCL* | *Estimate* | *LCL* | *UCL* | *Estimate* | *LCL* | *UCL* |
| **Additional month of paid leave** |  |  |  |  |  |  |  |  |  |  |  |  |
| Lagged three years, t-3 |  |  |  |  |  |  | -2.5 | -7.6 | 2.6 |  |  |  |
| Lagged two years, t-2 |  |  |  |  |  |  | -1.7 | -11.0 | 7.6 |  |  |  |
| **Lagged one year, t-1** | **-3.6** | **-9.6** | **2.4** | **-10.3** | **-15.7** | **-4.9** | **-4.4** | **-7.2** | **-1.6** | **-10.1** | **-16.5** | **-3.8** |
| Concurrent, t |  |  |  |  |  |  | -3.4 | -8.7 | 2.0 |  |  |  |
| Lead, t+1 |  |  |  |  |  |  | 0.6 | -8.9 | 10.0 |  |  |  |
| Lead two years, t+2 |  |  |  |  |  |  | -1.6 | -9.1 | 5.9 |  |  |  |
| Lead three years, t+3 |  |  |  |  |  |  | -1.7 | -10.1 | 6.8 |  |  |  |
|  | *Individual and household-level covariates^f^* | | | | | | | | | | | |
| Male gender | 9.8 | 7.3 | 12.3 | 10.3 | 7.6 | 13.0 | 9.8 | 7.3 | 12.3 | 9.0 | 6.0 | 12.0 |
| Mother's education (years) | -1.5 | -3.0 | -0.1 | -1.1 | -1.5 | -0.6 | -1.5 | -3.0 | -0.1 | -2.0 | -4.0 | 0.1 |
| 2nd wealth quintile | -4.4 | -11.3 | 2.4 | -0.8 | -4.4 | 2.8 | -4.4 | -11.3 | 2.4 | -7.4 | -14.3 | -0.4 |
| 3rd wealth quintile | -2.2 | -9.6 | 5.2 | -2.9 | -7.0 | 1.1 | -2.2 | -9.6 | 5.3 | 0.7 | -9.0 | 10.4 |
| 4th wealth quintile | -9.4 | -13.0 | -5.8 | -5.6 | -10.7 | -0.5 | -9.4 | -13.0 | -5.8 | -8.8 | -10.7 | -6.9 |
| 5th quintile (highest) | -13.6 | -20.0 | -7.1 | -11.5 | -18.9 | -4.2 | -13.6 | -20.0 | -7.1 | -10.6 | -15.7 | -5.6 |
| Urban residence | 0.4 | -5.2 | 6.0 | 0.5 | -4.2 | 5.3 | 0.4 | -5.2 | 6.0 | 4.1 | -0.3 | 8.5 |
| Short birth interval | 32.9 | 22.7 | 43.1 | 32.6 | 24.2 | 40.9 | 32.9 | 22.7 | 43.1 | 31.5 | 20.2 | 42.7 |
| Maternal age 20-39 | -24.0 | -31.0 | -16.9 | -20.5 | -28.6 | -12.3 | -24.0 | -31.0 | -16.9 | -22.0 | -32.3 | -11.7 |
| Maternal age >=40 | -6.5 | -17.2 | 4.3 | -5.8 | -17.2 | 5.6 | -6.5 | -17.2 | 4.2 | -11.5 | -29.2 | 6.2 |
| Skilled attendant at delivery | -1.1 | -9.4 | 7.1 | -6.9 | -11.1 | -2.7 | -1.1 | -9.2 | 6.9 | 2.4 | -9.5 | 14.3 |
|  | *Country-level covariates* | | | | | | | | | | | |
| Wage replacement rate |  |  |  | 0.1 | 0.0 | 0.2 | 0.1 | -0.1 | 0.3 | 0.3 | 0.0 | 0.7 |
| ln GDP per capita | -15.7 | -90.5 | 59.1 | -22.6 | -67.5 | 22.4 | -5.4 | -103.3 | 92.5 | 10.4 | -77.1 | 97.9 |
| female labor force participation | 0.4 | -0.8 | 1.6 | -0.4 | -1.2 | 0.5 | 0.5 | -0.5 | 1.5 | 1.3 | -0.9 | 3.5 |
| ln government health expenditure | -3.8 | -14.2 | 6.5 | -4.2 | -17.3 | 9.0 | -10.0 | -21.0 | 1.1 | -13.6 | -31.7 | 4.5 |
| ln total health expenditure | -17.5 | -37.4 | 2.5 | -16.2 | -35.5 | 3.2 | -11.8 | -34.1 | 10.5 | 0.5 | -28.7 | 29.6 |
| Sample size | 274716 | | | 274716 | | | 274716 | | | 274716 | | |

^a^ Model (a) measures the effects of an additional month of paid leave in full time equivalent (FTE) units, obtained by multiplying the legislated length of leave by the wage replacement rate (therefore this model does not additionally control for the wage replacement rate)

^b^ Model (b) provides unweighted estimates

^c^ Model (c) added to our primary exposure, the lagged effect of paid maternity leave (t-1), parameters representing paid maternity leave in preceding years (t-3, t-2), the survey year (t), and subsequent years (t+1, t+2, t+3); this model examines if there were persistent effects of the policy changes (t-3, t-2), and whether effects of policy changes that occurred during or after the measurement of our outcomes were observed (t, t+1, t+2, t+3)

^d^ Model (d) restricted the set of control countries to those with similar trends in infant mortality in the decade prior to the study period, as described in the S2 Text

^e^ LCL and UCL indicate lower and upper limits of the 95% confidence interval, respectively

^f^ Reference categories for categorical variables are female (vs. male) gender, the 1^st^ (lowest) wealth quintile, rural (vs. urban) residence, longer (vs. <24 month) birth interval, lower (<20 year) maternal age, and absence (vs. presence) or a skilled attendant at delivery
