# Supplementary material for: Increased Duration of Paid Maternity Leave Lowers Infant Mortality in Low- and Middle-Income Countries: A Quasi-Experimental Study
Source: PLoS Med. 2016 Mar 29;13(3):e1001985. doi: 10.1371/journal.pmed.1001985 (PMC4811564; doi:10.1371/journal.pmed.1001985)
Supplement: S1 Text — Checklist of items, extended from the STROBE statement, that should be reported in observational studies using routinely collected health data. (DOCX) [file pmed.1001985.s011.docx]

**The RECORD statement – checklist of items, extended from the STROBE statement, that should be reported in observational studies using routinely collected health data.**

|  | **Item No.** | **STROBE items** | **Location in manuscript where items are reported** | **RECORD items** | **Location in manuscript where items are reported** | **Details/extracts from manuscript** |
| --- | --- | --- | --- | --- | --- | --- |
| **Title and abstract** | | | | | |  |
|  | 1 | (a) Indicate the study’s design with a commonly used term in the title or the abstract (b) Provide in the abstract an informative and balanced summary of what was done and what was found | Abstract and Methods; Table 1 | RECORD 1.1: The type of data used should be specified in the title or abstract. When possible, the name of the databases used should be included.  RECORD 1.2: If applicable, the geographic region and timeframe within which the study took place should be reported in the title or abstract.  RECORD 1.3: If linkage between databases was conducted for the study, this should be clearly stated in the title or abstract. | RECORD 1.1: The type of data and the name of the databases used are provided in the Abstract, as well as the Methods (Sample) section  RECORD 1.2: The geographic regions and timeframe are provided in the Abstract and Methods (Sample) sections, as well as Table 1  RECORD 1.3: The linkage between the databases used to measure exposures and outcomes is described in the Methods section | In this study, we utilized a difference-in-differences approach to provide the first evaluation of whether paid maternity leave policies affect infant mortality in LMICs.  We used birth history data collected via the Demographic and Health Surveys to assemble a panel of approximately 300,000 live births in 20 countries from 2000–2008; these observational data were merged with longitudinal information on the duration of paid maternity leave provided by each country. We estimated the effect of an increase in maternity leave in the prior year on the probability of infant (<1 year), neonatal (< 28 days), and post-neonatal (between 28 days and 1 year after birth) mortality. Fixed effects for country and year were included to control for, respectively, unobserved time-invariant confounders that varied across countries and temporal trends in mortality that were shared across countries. Average rates of infant, neonatal, and post-neonatal mortality over the study period were 55.2, 30.7, and 23.0 per 1000 live births, respectively. Each additional month of paid maternity leave led to 7.9 fewer infant deaths per 1000 live births (95% confidence interval (CI)=3.7, 12.0), reflecting a 13% relative reduction. Reductions in infant mortality resulting from increases in the duration of paid maternity leave were concentrated in the post-neonatal period. Estimates were robust to adjustment for individual, household, and country-level confounders, although there may be residual confounding by unmeasured time-varying confounders, such as coincident policy changes. |
| **Introduction** | | | | | |  |
| Background rationale | 2 | Explain the scientific background and rationale for the investigation being reported | Background |  |  | A multidisciplinary literature suggests paid maternity and parental leave policies are consistently associated with improvements in child health, including reductions in neonatal and infant mortality, in high-income countries.[1-5] Paid maternity leave may affect neonatal and infant mortality through several mechanisms. However, the population health effects of maternity leave policies in poorer countries have not been examined. This is the first multilevel study to assess whether maternity leave policies affect infant health in low- and middle-income countries (LMICs). |
| Objectives | 3 | State specific objectives, including any prespecified hypotheses | last paragraph of Background |  |  | We utilized a difference-in-differences approach to estimate the influence of paid maternity leave on neonatal and infant mortality. |
| **Methods** | | | | | |  |
| Study Design | 4 | Present key elements of study design early in the paper | last paragraph of Background |  |  | We constructed a database that measures maternity leave policies for LMICs over time, merged this information to a sample of live births occurring between 2000 and 2008 in 20 LMICs surveyed as part of the Demographic and Health Surveys (DHS), and utilized a difference-in-differences approach to estimate the influence of paid maternity leave on neonatal and infant mortality. |
| Setting | 5 | Describe the setting, locations, and relevant dates, including periods of recruitment, exposure, follow-up, and data collection | Methods (Sample); Table 1 |  |  | Information on birth outcomes was derived from the DHS. The DHS collect comparable information on demographic, socioeconomic, nutritional, behavioral, fertility and health characteristics from a nationally representative sample of households in LMICs using a two-stage cluster sampling design. Individuals are selected for interviews from the household roster; information is collected on women of reproductive age (15-49 years), men (usually aged 15-54 or 15-59), and children under the age of five years. Trained interviewers and standardized tools and measurement techniques are used to ensure comparability of surveys across countries and survey waves. Further details regarding sampling strategies and study procedures are available elsewhere.^21,22^  Table 1 reports survey years and sample sizes for the sampled countries. |
| Participants | 6 | *(a) Cohort study* - Give the eligibility criteria, and the sources and methods of selection of participants. Describe methods of follow-up  *Case-control study* - Give the eligibility criteria, and the sources and methods of case ascertainment and control selection. Give the rationale for the choice of cases and controls  *Cross-sectional study* - Give the eligibility criteria, and the sources and methods of selection of participants  *(b) Cohort study* - For matched studies, give matching criteria and number of exposed and unexposed  *Case-control study* - For matched studies, give matching criteria and the number of controls per case | Methods (Sample); Table 1 | RECORD 6.1: The methods of study population selection (such as codes or algorithms used to identify subjects) should be listed in detail. If this is not possible, an explanation should be provided.  RECORD 6.2: Any validation studies of the codes or algorithms used to select the population should be referenced. If validation was conducted for this study and not published elsewhere, detailed methods and results should be provided.  RECORD 6.3: If the study involved linkage of databases, consider use of a flow diagram or other graphical display to demonstrate the data linkage process, including the number of individuals with linked data at each stage. | RECORD 6.1: Details of sample selection are provided in Methods (Sample)  RECORD 6.2: Not applicable  RECORD 6.3: The linkage, a simple merge of policy (exposure) data to serial cross-sections from the Demographic and Health Surveys, is explained in the Methods section | Mothers surveyed in the DHS were asked to provide information concerning all children born alive in the previous five years. These data were used to construct a panel of live births, each with information on vital status, over a consistent set of years and countries. We created two separate samples, one for our analyses of infant and post-neonatal mortality and the other for analyses of neonatal mortality. We restricted these samples to the 282,836 and 304,294 live births that occurred at least one year and at least 28 days prior to the DHS interview date, respectively, in order to ascertain whether each child survived the infant (1 year) and neonatal (28 day) periods following birth. After further excluding observations with missing information on key covariates, our samples were composed of 282,751 live births between 2000-2007 for analyses of infant and post-neonatal mortality and 304,201 live births between 2001-2008 for analyses of neonatal mortality.  Table 1 reports survey years and sample sizes for the sampled countries. |
| Variables | 7 | Clearly define all outcomes, exposures, predictors, potential confounders, and effect modifiers. Give diagnostic criteria, if applicable. | Methods (Measures) | RECORD 7.1: A complete list of codes and algorithms used to classify exposures, outcomes, confounders, and effect modifiers should be provided. If these cannot be reported, an explanation should be provided. | The precise definitions or exposures, outcomes, and confounders are provided in the Methods (Measures) | The exposure of interest in our study was the legislated length of paid maternity leave for each country and year. Data on current maternity leave policies for each sampled country were provided by UCLA’s World Legal Rights Data Centre (WoRLD) and then collected retrospectively to 1995 by McGill University’s Maternal and Child Health Equity (MACHEquity) research program. We defined the duration of paid maternity leave as the legislated length of paid leave available to mothers only. We did not distinguish leave that could be taken in the prenatal period from leave that could be taken after birth; however, few LMICs make an explicit distinction between prenatal and postnatal leave as mandatory. Further details regarding the collection and coding of global maternity leave policies is available elsewhere.^1^  The outcome variables, infant, neonatal, and post-neonatal mortality, were measured using the five-year birth histories provided by women interviewed in the DHS. We created binary indicators for infant, neonatal, and post-neonatal mortality to measure whether each child died within one year, within the first 28 days of life, or between 28 days and 1 year after birth, respectively. |
| Data sources/ measurement | 8 | For each variable of interest, give sources of data and details of methods of assessment (measurement).  Describe comparability of assessment methods if there is more than one group | Methods (Measures) |  |  | Outcome: The outcome variables, infant, neonatal, and post-neonatal mortality, were measured using the five-year birth histories provided by women interviewed in the DHS. We created binary indicators for infant, neonatal, and post-neonatal mortality to measure whether each child died within one year, within the first 28 days of life, or between 28 days and 1 year a  Exposure: The exposure of interest in our study was the legislated length of paid maternity leave for each country and year. Data on current maternity leave policies for each sampled country were provided by UCLA’s World Legal Rights Data Centre (WoRLD) and then collected retrospectively to 1995 by McGill University’s Maternal and Child Health Equity (MACHEquity) research program.fter birth, respectively.  Covaraites: We collected information on potentially confounding individual, household, and country-level characteristics based on a review of the literature on determinants of neonatal and infant mortality in LMICs.^23-31^ Individual and household-level socio-demographic characteristics included the child’s gender, mother’s educational attainment in completed years, urban/rural residence, and household socioeconomic status (SES). Household SES, categorized into quintiles for analysis, was measured using a wealth index provided by the DHS, which is based on ownership of specific assets (e.g., bicycle, radio and television), environmental conditions, and housing characteristics (e.g., type of water source, sanitation facilities, materials used for housing construction), and constructed using the method proposed by Filmer and Pritchett.^32,33^ Birth characteristics included the interval between births (defined as short if it was less than 24 months), maternal age at the time of each birth (categorized as <20 years, 20-39, or 40 and older), and whether there was a skilled birth attendant present at the time of delivery. Aside from the length of paid leave, another defining feature of paid maternity leave policies is the percentage at which wages are replaced. Because changes in the wage replacement rate sometimes coincided (i.e., in Lesotho) with changes in the legislated length of leave, we controlled for the wage replacement rate as a potential confounder. Additional time-varying country-level covariates included levels of economic development (measured by GDP per capita, purchasing power parity, constant 2005 international dollars), levels of female labor force participation, per capita total health expenditure, and per capita government health expenditure, which were available from the World Bank's World Development Indicators and Global Development Finance (WDI and GDF) databases.^34^ |
| Bias | 9 | Describe any efforts to address potential sources of bias | Statistical analyses; Sensitivity analyses |  |  | Using our difference-in-differences design, the effects of paid maternity leave policies were identified by changes in outcomes occurring within treated countries that modified their policies relative to corresponding changes in control countries that did not modify their policies. We included fixed effects for country ($\alpha_{j}$) and year ($\lambda_{t}$) to control for unobserved time-invariant confounders that vary across countries, and any temporal trends in mortality that are shared across countries, respectively. We additionally controlled for measured individual, household, and country-level characteristics, including the lagged wage replacement rate, per capita GDP, female labor force participation, and per capita total and government health expenditures. Therefore, any unmeasured confounders that would remain to bias our estimates would have to coincide with policy changes occurring within treated countries and also influence mortality, which markedly reduces the list of potential unobserved confounders.  We also conducted a number of sensitivity analyses and robustness checks, which are described in the manuscript and appendix. |
| Study size | 10 | Explain how the study size was arrived at | Methods (Sample); Table 1 |  |  | Our sample was comprised of all live births occurring to DHS respondents from 20 LMICs between 2000 and 2008. These countries were selected because they administered at least two DHS surveys between 2001 and 2011, which permitted analyses of policy changes occurring within countries over time. Briefly, mothers surveyed in the DHS were asked to provide information concerning all children born alive in the previous five years. These data were used to construct a panel of live births, each with information on vital status, over a consistent set of years and countries. We created two separate samples, one for our analyses of infant and post-neonatal mortality and the other for analyses of neonatal mortality. We restricted these samples to the 282,836 and 304,294 live births that occurred at least one year and at least 28 days prior to the DHS interview date, respectively, in order to ascertain whether each child survived the infant (1 year) and neonatal (28 day) periods following birth. After further excluding observations with missing information on key covariates, our samples were composed of 282,751 live births between 2000-2007 for analyses of infant and post-neonatal mortality and 304,201 live births between 2001-2008 for analyses of neonatal mortality. **Table 1** reports survey years and sample sizes for the sampled countries. |
| Quantitative variables | 11 | Explain how quantitative variables were handled in the analyses. If applicable, describe which groupings were chosen, and why | Methods (Measures) |  |  | See Item No. 8 |
| Statistical methods | 12 | (a) Describe all statistical methods, including those used to control for confounding  (b) Describe any methods used to examine subgroups and interactions  (c) Explain how missing data were addressed  (d) *Cohort study* - If applicable, explain how loss to follow-up was addressed  *Case-control study* - If applicable, explain how matching of cases and controls was addressed  *Cross-sectional study* - If applicable, describe analytical methods taking account of sampling strategy  (e) Describe any sensitivity analyses | Methods (Statistical analyses) |  |  | We linked data on paid maternity leave policies to outcomes and covariates from the DHS by country-year and examined the effects of an increase in the duration of paid maternity leave on the risk of mortality during the first year of life. Specifically, we estimated for birth $i$ the effect of an additional month of paid leave (lagged by one year to respect the temporal ordering between the policy and outcome) on the probabilities of infant, neonatal, and post-neonatal mortality using a linear probability models of the general form: $Y_{ijt}=\alpha_{j}+\beta M_{jt-1}+\lambda_{t}+\epsilon_{ijt}$, where $\beta$ measures the effect of a one-month increase in maternity leave in the prior year, $M_{jt-1}$(where *j* indexes the country and *t* the year), on infant, neonatal, and post-neonatal death, $Y_{\mathrm{ijt}}$. We included fixed effects for country ($\alpha_{j}$) and year ($\lambda_{t}$) to control for unobserved time-invariant confounders that vary across countries, and any temporal trends in mortality that are shared across countries, respectively. The effects of paid maternity leave policies were therefore identified by changes in outcomes occurring within treated countries that modified their policies relative to corresponding changes in control countries that did not modify their policies.  In the first model, we estimated the effect of an additional month of paid maternity leave on mortality after including country and year fixed effects (Model 1). Second, we additionally controlled for measured individual, household, and country-level characteristics, including the lagged wage replacement rate, per capita GDP and female labor force participation (Model 2). Third, we included controls for per capita total and government health expenditures (Model 3); these data were unavailable for all years for Zimbabwe and observations from Zimbabwe were therefore dropped from Model 3 analyses. In order to examine potential non-linearity in the effect of paid maternity leave, we introduced a quadratic duration of paid leave variable to our fully adjusted model (Model 3). We also estimated the fully adjusted model on the risk ratio scale. All analyses incorporated respondent-level sampling weights to account for individual survey sample designs. Per DHS guidelines, we used information on the number of women aged 15-49 in each survey-year, provided by the Population Division of the United Nations,^35^ and applied the de-normalization of standard weights approach described in the DHS Sampling and Household Listing Manual,^36^ in order to calculate an appropriate weight for each observation in the analyses. All models incorporated robust standard errors to account for clustering at the country level.^37^ |
| Data access and cleaning methods |  | .. |  | RECORD 12.1: Authors should describe the extent to which the investigators had access to the database population used to create the study population.  RECORD 12.2: Authors should provide information on the data cleaning methods used in the study. | RECORD 12.1: Methods (Sample)  RECORD 12.2: Methods (Sample and Measures) | RECORD 12.1: The authors had full access to the Demographic and Health Survey data from which the analytic sample is derived.  RECORD 12.2: An overview of the process used to derive our analytic dataset is described in the Methods section. Additionally, the code use to clean and prepare our analytic dataset will be made publicly available at the time of publication. |
| Linkage |  | .. |  | RECORD 12.3: State whether the study included person-level, institutional-level, or other data linkage across two or more databases. The methods of linkage and methods of linkage quality evaluation should be provided. | RECORD 12.3: The linkage between the databases used to measure exposures and outcomes is described in the Methods section | RECORD 12.3: We linked data on paid maternity leave policies to outcomes and covariates from the DHS by country-year and examined the effects of an increase in the duration of paid maternity leave on the risk of mortality during the first year of life. |
| **Results** | | | | | |  |
| Participants | 13 | (a) Report the numbers of individuals at each stage of the study (*e.g.*, numbers potentially eligible, examined for eligibility, confirmed eligible, included in the study, completing follow-up, and analysed)  (b) Give reasons for non-participation at each stage.  (c) Consider use of a flow diagram | Methods (Sample); Table 1 | RECORD 13.1: Describe in detail the selection of the persons included in the study (*i.e.,* study population selection) including filtering based on data quality, data availability and linkage. The selection of included persons can be described in the text and/or by means of the study flow diagram. |  | (a) See Item No. 6  (b) Missing observations (<0.1% of the data) were excluded. All analyses incorporated respondent-level sampling weights to account for individual survey sample designs. Per DHS guidelines, we used information on the number of women aged 15-49 in each survey-year, provided by the Population Division of the United Nations,[19] and applied the de-normalization of standard weights approach described in the DHS Sampling and Household Listing Manual,[20] in order to calculate an appropriate weight for each observation in the analyses. |
| Descriptive data | 14 | (a) Give characteristics of study participants (*e.g.*, demographic, clinical, social) and information on exposures and potential confounders  (b) Indicate the number of participants with missing data for each variable of interest  (c) *Cohort study* - summarise follow-up time (*e.g.*, average and total amount) | Results; Methods (Sample); Table 1; Table S1, Table 2, Figure S1 |  |  | (a) Table 1 shows the rates of neonatal and infant mortality for the 20 DHS countries; average rates of neonatal and infant mortality over the study period were 30.7 and 55.2 per 1000 live births, respectively. eTable 1 provides baseline values of key covariates for treated and control countries. Table 2 shows trends in paid maternity leave benefits and country-level characteristics for the five treated and 15 control countries; trends in the duration of paid leave for individual treated countries are shown in eFigure 1. For treated countries, the average length of paid leave increased from 7.6 (SD=5.4) weeks in 2000 to 12.2 (SD=3.8) weeks in 2008. The average length of paid leave in control countries was 12.2 (SD=3.0) weeks.  (b) As noted earlier, very few (<0.1%) of observations were missing.  (c) Our analyses were based on representative serial cross-sectional samples. |
| Outcome data | 15 | *Cohort study* - Report numbers of outcome events or summary measures over time  *Case-control study* - Report numbers in each exposure category, or summary measures of exposure  *Cross-sectional study* - Report numbers of outcome events or summary measures | Results; Table 1 |  |  | Table 1 shows the rates of neonatal and infant mortality for the 20 DHS countries; average rates of neonatal and infant mortality over the study period were 30.7 and 55.2 per 1000 live births, respectively. eTable 1 provides baseline values of key covariates for treated and control countries. |
| Main results | 16 | (a) Give unadjusted estimates and, if applicable, confounder-adjusted estimates and their precision (e.g., 95% confidence interval). Make clear which confounders were adjusted for and why they were included  (b) Report category boundaries when continuous variables were categorized  (c) If relevant, consider translating estimates of relative risk into absolute risk for a meaningful time period | Methods (Measures); Methods (Statistical analyses); Results); Table 3, Table 4, Table S2 |  |  | (a) Tables 3, 4, and 5 show the unadjusted and adjusted effects on the probability of infant, neonatal, and post-neonatal death, respectively, of an additional month of paid maternity leave.  (b) Household SES, split into quintiles for analysis, was measured using a wealth index provided by the DHS, which is based on ownership of specific assets (e.g., bicycle, radio and television), environmental conditions, and housing characteristics (e.g., type of water source, sanitation facilities, materials used for housing construction), and constructed using the method proposed by Filmer and Pritchett.[17, 18] Birth characteristics included the interval between births (defined as short if it was less than 24 months) and maternal age at the time of each birth (categorized as <20 years, 20-39, or 40 and older).  (c) For the main results, estimates were provided on the risk difference or probability scale. Risk ratio estimates were also provided in eTable 2. |
| Other analyses | 17 | Report other analyses done—e.g., analyses of subgroups and interactions, and sensitivity analyses | Methods (Sensitivity analyses); S2 Text |  |  | We conducted sensitivity analyses to assess the robustness of our main findings. First, because there is no standard metric for maternity leave policies, we measured the effects of paid leave in full time equivalent (FTE) units, obtained by multiplying the legislated length of leave by the wage replacement rate, in addition to the duration of any paid leave. Further details regarding the calculation of FTE units of paid leave are available elsewhere.^1,16^ Second, we examined whether results were sensitive to the inclusion of sampling weights. Third, we added to our primary exposure, the lagged effect of paid maternity leave (t-1), parameters representing paid maternity leave in preceding years (t-3, t-2), the survey year (t), and subsequent years (t+1, t+2, t+3). These analyses assessed if there were persistent effects of the policy changes, as well as whether observed effects were responses to policy changes that occurred in the period during or before the measurement of our outcomes, as we would expect if the effects of paid maternity leave policies were causal. Fourth, as described in the **Appendix**, we assessed whether our findings were sensitive to the particular selection of control countries. In these analyses, we examined trends in infant, neonatal, and post-neonatal mortality occurring in the decade before our treated countries changed their legislated length of paid maternity leave and then restricted the sample of controls to those countries with parallel pre-intervention trends.  Also see the Appendix. |
| **Discussion** | | | | | |  |
| Key results | 18 | Summarise key results with reference to study objectives | Discussion |  |  | We merged longitudinal information on the legislated duration of paid maternity leave with a panel of approximately 300,000 live births that occurred in 20 DHS countries between 2000–2008 and used this dataset to conduct the first evaluation of the impact of paid maternity leave on infant mortality in LMICs. Difference-in-differences analyses suggested that each additional month of paid maternal leave led to approximately 8 fewer infant deaths per 1000 live births. These findings were relatively robust to alternative model specifications. |
| Limitations | 19 | Discuss limitations of the study, taking into account sources of potential bias or imprecision. Discuss both direction and magnitude of any potential bias | Discussion | RECORD 19.1: Discuss the implications of using data that were not created or collected to answer the specific research question(s). Include discussion of misclassification bias, unmeasured confounding, missing data, and changing eligibility over time, as they pertain to the study being reported. | RECORD 19.1: We discussed these implications in the Limitations section of our Discussion and elaborated on the key identifying assumptions underlying our approach in S2 Text. | There were limitations to our study. First, as in any non-experimental study, there is the potential for unmeasured confounding. However, we controlled for potential confounding by individual, household, and country-level characteristics. We also included fixed effects for country and year to account for unobserved time-invariant confounders that vary across countries and any temporal trends in mortality that are shared across countries, respectively. Therefore, any unmeasured confounders that would remain to bias our estimates would have to coincide with policy changes occurring within treated countries and also influence mortality, which markedly reduces the list of potential unobserved confounders. However, the adoption of several policies, including paid leave policies, simultaneously would conflate effects and potentially bias our results. Second, the determination of our outcomes, neonatal and infant mortality, depends on maternal recall and mothers may underreport the births and deaths of children who are not alive at the time of interview. Such underreporting would bias our estimates only if it were differential between our treatment and control countries. Third, our measure of paid maternity leave is calculated based on the legislated maternity leave and does not account for other leave (i.e., parental leave) that might also be available to mothers. Fourth, in order to model the longitudinal effect of maternity leave policies on neonatal and infant mortality we limited our analyses to 20 selected LMICs with at least two DHS surveys between 2001 and 2011; the inclusion of sampling weights allows us to generalize our results to these 20 countries, but not to all LMICs.  See also the Appendix. |
| Interpretation | 20 | Give a cautious overall interpretation of results considering objectives, limitations, multiplicity of analyses, results from similar studies, and other relevant evidence | Discussion |  |  | Caveats considered, our analyses suggest that an additional month of paid maternity leave led to as much as a 13% reduction in infant mortality. From a policy planning perspective, further work is needed to elucidate the mechanisms that explain the benefits of paid maternity leave on infant mortality and evaluate the optimal balance of leave from employment prior to and following delivery, as well as varying levels of compensation. Further work is also needed to document the effects of paid maternity leave on women’s labor force participation, health, and well-being in LMICs. |
| Generalisability | 21 | Discuss the generalisability (external validity) of the study results |  |  |  |  |
| **Other Information** | | | | | |  |
| Funding | 22 | Give the source of funding and the role of the funders for the present study and, if applicable, for the original study on which the present article is based | Discussion |  |  | In order to model the longitudinal effect of maternity leave policies on neonatal and infant mortality we limited our analyses to 20 selected LMICs with at least two DHS surveys between 2001 and 2011; the inclusion of sampling weights allows us to generalize our results to these 20 countries, but not to all LMICs. |
| Accessibility of protocol, raw data, and programming code |  | .. |  | RECORD 22.1: Authors should provide information on how to access any supplemental information such as the study protocol, raw data, or programming code. | RECORD 22.1: We have included a Data Availability Statement | Analyses were based on two sources of publicly available data, specifically: (1) live births information collected from respondents in 20 countries (Table 1) surveyed as part of the Demographic and Health Surveys (DHS) and (2) measures of maternity leave policies. The DHS data are publicly available, but users must first register with the DHS program. Registration, which requires a summary of the proposed study and selection of country datasets, can be completed at: <http://www.dhsprogram.com/data/new-user-registration.cfm>. Data on current maternity leave policies for each sampled country were provided by UCLA’s World Legal Rights Data Centre (WoRLD) and then collected retrospectively to 1995 by McGill University’s Maternal and Child Health Equity (MACHEquity) research program. The policy data are freely available through the website, [www.machequity.com](http://www.machequity.com), without any restriction. The policy data, as well as the statistical code for producing the analytic dataset and replicating our results (given DHS data that must be downloaded from the DHS program), are also available with unrestricted access from the corresponding author’s Dataverse: <https://dataverse.harvard.edu/dataverse/harvard>. |

*Reference: Benchimol EI, Smeeth L, Guttmann A, Harron K, Moher D, Petersen I, Sørensen HT, von Elm E, Langan SM, the RECORD Working Committee. The REporting of studies Conducted using Observational Routinely-collected health Data (RECORD) Statement. *PLoS Medicine* 2015; in press.

*Checklist is protected under Creative Commons Attribution ([CC BY](http://creativecommons.org/licenses/by/4.0/)) license.
