# Supplementary material for: Increased Duration of Paid Maternity Leave Lowers Infant Mortality in Low- and Middle-Income Countries: A Quasi-Experimental Study
Source: PLoS Med. 2016 Mar 29;13(3):e1001985. doi: 10.1371/journal.pmed.1001985 (PMC4811564; doi:10.1371/journal.pmed.1001985)
Supplement: S2 Text — (DOCX) [file pmed.1001985.s012.docx]

**S2 TEXT. Examination of pre-intervention trends**

The difference-in-differences approach provides an unbiased causal effect if the control units included represent the change in the outcome that would have occurred in the treated units absent the treatment—in our case, this would be true if the change in mortality in control countries that did not change their maternity leave policies during the study period was equivalent to the change that would have occurred in treated countries that did, *had they not changed their policies*. This assumption of “parallel trends” is more difficult to check visually in the fixed effects generalization of the difference-in-differences approach, with multiple treated and control countries and several policy changes. However, the observation of similar trends in outcomes in the pre-intervention period lends some assurance that this assumption might not be violated.

We lacked time-series data from the Demographic Health Surveys (DHS) showing pre-intervention trends in our outcomes for all of our sampled countries. In lieu of the individual-level data from the DHS, we obtained information on the infant, neonatal, and post-neonatal mortality rates (per 1000 live births), as well as the absolute number of deaths, that occurred in our sampled countries in the decade (i.e., 1990-2000) before our treated countries began to adopt the policy changes that we exploited in our analyses; these data are available through the World Bank's World Development Indicators database. We plotted the trends in mortality rates (per 1000 live births) between 1990 and 2000 for treated (i.e., Bangladesh, Kenya, Lesotho, Uganda, Zimbabwe) and control (i.e., Armenia, Bolivia, Colombia, Egypt, Ghana, Honduras, Cambodia, Madagascar, Malawi, Nigeria, Nepal, Philippines, Rwanda, Senegal, Tanzania) countries weighted by the number of deaths that occurred in each country and year. As illustrated by **S3 Figure**, there was some evidence that the pre-intervention trends in treated and control countries were not parallel, particularly for neonatal mortality, where trend lines actually intersected in the late 1990s. By contrast, trends in post-neonatal mortality were roughly parallel for treated and control countries.

To examine the sensitivity of our findings to the selection of control countries, and particularly the inclusion of control countries that may have misrepresented the counterfactual trends in mortality that we were attempting to recover, we restricted our sample of control countries to those with similar pre-intervention trends between 1990 and 2000. For analyses of infant mortality, we eliminated control countries from the analysis if the change in the infant mortality rate between 1990 and 2000 deviated from the change in the infant mortality rate for the five treated countries by more than 10 deaths per 1000 live births; there were seven remaining control countries (i.e., Armenia, Bolivia, Egypt, Ghana, Honduras, Madagascar, Tanzania). For analyses of neonatal mortality, we eliminated control countries from the analysis if the change in the neonatal mortality rate between 1990 and 2000 deviated from the change in the neonatal mortality rate for the five treated countries by more than 5 deaths per 1000 live births, leaving eight control countries (i.e., Armenia, Bolivia, Egypt, Honduras, Madagascar, Malawi, Nepal, Tanzania). As pre-intervention trends in post-neonatal mortality appeared relatively parallel, we did not eliminate any control countries for analysis of this outcome.

As illustrated by **S4 Figure**, the pre-intervention trends in treated and control countries were roughly parallel after we imposed these criteria. We then estimated the effects of an increase in paid maternity leave on the number of infant and neonatal deaths per 1000 live births in samples comprised of the treated countries and selected controls. For both infant (**S4 Table, Model d**) and neonatal (**S5 Table, Model d**) mortality, our estimates were qualitatively similar to our main findings utilizing all controls, suggesting our findings were fairly robust to the selection of controls.
