# Supplementary material for: Increased Duration of Paid Maternity Leave Lowers Infant Mortality in Low- and Middle-Income Countries: A Quasi-Experimental Study
Source: PLoS Med. 2016 Mar 29;13(3):e1001985. doi: 10.1371/journal.pmed.1001985 (PMC4811564; doi:10.1371/journal.pmed.1001985)
Supplement: S3 Text — (DOCX) [file pmed.1001985.s013.docx]

**S3 TEXT. Analysis history**

This study is part of a larger program of work focused on estimating the impact of social policies on health outcomes prioritized by the United Nations Millennium Development Goals (further information available at <http://machequity.com/>). This research is based on the analysis of secondary, de-identified data. The Institution Review Board of McGill University’s Faculty of Medicine provided ethics approval. A summary of our overall research objective was also submitted to Measure DHS (<http://www.dhsprogram.com/>) in order to gain access to Demographic and Health Survey data. We did not pre-register or publish the protocol for this particular analysis of maternity leave policies and infant mortality. We outline our hypotheses, as well as our sampling and statistical strategies below, highlighting areas where we modified our approach after the study had commenced.

**Hypotheses**

As described in the main text (Background), evidence from high-income contexts suggests that more generous maternity and parental leave policies are associated with lower rates of infant mortality. Based on our review of the literature, we posited that increases in maternity benefits would be associated with lower rates of mortality in the first year of life. However, our priors were fairly uninformative given the lack of credible evidence from low- and middle-income contexts, as well as theory suggesting that the effect of leave policies may vary in lower-income (vs. higher-income) contexts, given differences in the nature of non-parental child-care options as well as of women’s participation in the formal economy.

**Sample selection**

The inclusion criteria were decided in advance of the analyses and were not modified. At the country-level, we decided *a priori* which countries and years would be included in our analysis and the selection of country-years has not changed since we conceived of and designed our study. Our selection of countries was informed by the availability of two types of data: (1) cross-national information on maternity leave policies, and (2) data on the vital status of live births occurring in low- and middle-income countries (LMICs) as captured by the Demographic and Health Surveys (DHS). As part of a broader initiative, we have collected standardized information on paid maternity leave policies in low- and middle-income countries between 1995-2013, as described in the manuscript. We wanted to join this information on national maternity leave policies to outcomes for births captured by available Demographic and Health Surveys (DHS) in manner that would allow us to estimate the impact of changes in maternity leave policies on the probabilities infant and neonatal mortality.

Mothers who are surveyed as part of the DHS are asked about their five-year birth histories and this information can be used to assemble a serial cross-sectional panel of live births occurring in different LMICs in different years. For example, for a mother with reported births occurring three and five years ago at the time of survey in 2005, we can assign her two births to the years 2000 and 2002 and also record their vital status (i.e., did they survive past the neonatal and infant periods?). Using this approach, we mapped available DHS surveys from the most recent rounds since 2000 and identified a panel of 20 countries with information on live births occurring in every year between 2000 and 2008. We selected this period particularly because it included five countries that experienced policies changes. Alternatively, we could have selected fewer countries covering more years; however, this would have excluded treated countries (i.e., Kenya, Lesotho) that experienced a policy change. Thus, we struck a balance at 20 countries because it provided us with adequate policy variation and allowed us to have an uninterrupted, continuous panel of live births occurring over nearly a decade.

At the individual-level our sampling frame included all live births in the prior five years reported by mothers surveyed as part of the DHS. We restricted these samples to the 282,836 and 304,294 live births that occurred at least one year and at least 28 days prior to the DHS interview date, respectively, in order to ascertain whether each child survived the infant (1 year) and neonatal (28 day) periods following birth. The only other inclusion/exclusion criterion was to have non-missing information on key covariates (also decided *a priori*), which resulted in the loss of less than 0.1% of our data.

**Statistical analyses**

Our analysis plan, described in detail in the Methods section of the paper, was mostly decided *a priori* and followed a structure similar to prior published work by our group on maternity leave policies and vaccination coverage (i.e., see Hajizadeh et al., 2015). Nonetheless, there were some modifications to our analysis plan as our study progressed, including several additional analyses that were conducted in response to the thoughtful comments that we received during the review process. We summarize below the main *post hoc* modifications to our analysis plan.

1. Measurement of leave: There is no “gold standard” for measuring the generosity of maternity benefits available in a country. Two of the main dimensions of leave policies are the duration permitted and the wage replacement rate. Initially, we measured maternity benefits in full time equivalent (FTE) units, calculated by multiplying the duration of leave available by the wage replacement rate. However, one limitation of measuring benefits in FTE units relates to the interpretation of a unit-change, which could be due to a change in the duration of leave or the rate at which wages are replaced. To avoid potentially conflating the effect of changes in the duration of leave and the wage replacement rate, we decided to also measure the generosity of leave benefits by the duration of leave available, controlling for the wage replacement rate. Results from both analyses (with leave measured in FTE units and as the duration of leave controlling for the wage replacement rate) are provided in the paper.
2. Added post-neonatal mortality as an outcome: At the time of our first submission we examined the effects of paid maternity leave on neonatal (<28 day) and infant (<1 year) mortality. Given that the we observed beneficial effects of increased leave in the infant but not neonatal period, an astute reviewer suggested that we add post-neonatal mortality (death between 28 days and one year after birth) as an outcome, which we did in our subsequent revision. All three outcomes are now presented.
3. Investigation of non-linear effects: Our analysis plan initially specified a linear effect of the duration of paid leave on our outcomes. During the review process it was suggested that the impact of additional leave on infant mortality might be non-linear. Accordingly, we examined potential non-linearities in the effect of paid leave on our outcomes by introducing a quadratic duration of paid leave variable to our fully adjusted model in the revised version of the manuscript.
